# A double-hit of stress and low-grade inflammation on functional brain network mediates posttraumatic stress symptoms

**DOI:** 10.1038/s41467-020-15655-5

**Published:** 2020-04-20

**Authors:** Jungyoon Kim, Sujung Yoon, Suji Lee, Haejin Hong, Eunji Ha, Yoonji Joo, Eun Hee Lee, In Kyoon Lyoo

**Affiliations:** 1 Ewha Brain Institute, Ewha Womans University, Seoul, South Korea; 2 Department of Brain and Cognitive Sciences, Ewha Womans University, Seoul, South Korea; 3 Graduate School of Pharmaceutical Sciences, Ewha Womans University, Seoul, South Korea; 4 Green Cross Laboratories, Yongin, South Korea; 5 The Brain Institute and Department of Psychiatry, University of Utah, Salt Lake City, UT USA

**Keywords:** Network models, Stress and resilience, Post-traumatic stress disorder

## Abstract

Growing evidence indicates a reciprocal relationship between low-grade systemic inflammation and stress exposure towards increased vulnerability to neuropsychiatric disorders, including posttraumatic stress disorder (PTSD). However, the neural correlates of this reciprocity and their influence on the subsequent development of PTSD are largely unknown. Here we investigated alterations in functional connectivity among brain networks related to low-grade inflammation and stress exposure using two large independent data sets. Functional couplings among the higher-order cognitive network system including the salience, default mode, and central executive networks were reduced in association with low-grade inflammation and stress exposure. This reduced functional coupling may also be related to subsequent posttraumatic stress symptom severity. The current findings propose functional couplings among the higher-order cognitive network system as neural correlates of low-grade inflammation and stress exposure, and suggest that low-grade inflammation, alongside with stress, may render individuals more vulnerable to PTSD.

## Abstract (web summary)

Low-grade systemic inflammation and stress increase vulnerability to neuropsychiatric disorders. Here, the authors show that inflammation and stress-induced changes in higher order cognitive networks increase vulnerability to posttraumatic stress disorder.

## Introduction

Low-grade systemic inflammation is the chronic production of pro-inflammatory factors that may arise from persistent stressors to the body, including increased oxidative and psychosocial stress^1–3^. Growing evidence posits that the peripheral immune system can interact with the neurocircuitry involved in emotion regulation and behavior^4,5^, which may influence the onset of various neuropsychiatric disorders. Specifically, low-grade inflammation has shown to associate with increased risk of posttraumatic stress disorder (PTSD) and major depression^5–7^. Furthermore, continued exposure to psychosocial stress may reciprocally increase immune responses, leading to chronic low-grade inflammation^4,8^. Given that stress is a key precipitating factor in PTSD and major depression^9,10^, the interrelationship between low-grade inflammation and repeated stress exposure may play an important role in the development of subsequent clinical manifestations.

Previous neuroimaging studies have identified several brain regions that are sensitive to inflammation, including the prefrontal cortex, insula, anterior cingulate, limbic structures, and basal ganglia^4,5,11,12^. Notably, these regions are also known to undergo functional alteration in response to stress exposure^13,14^, and constitute much of the higher-order cognitive network system^15,16^. Recent studies have demonstrated that increased inflammation can alter functional connectivity of the major brain regions related to the development of anxiety in the case of major depression as well as those with comorbid PTSD^17^. Studies have also noted that altered functional connectivity due to increased inflammation correlates with the severity of clinical symptoms^17,18^. However, little is known about the potential role of these brain regions in the reciprocal interactions between low-grade inflammation and repeated stress exposure. Moreover, whether such brain alterations may be directly related to the development of subsequent psychopathology of PTSD remains inconclusive.

**Fig. 1:**
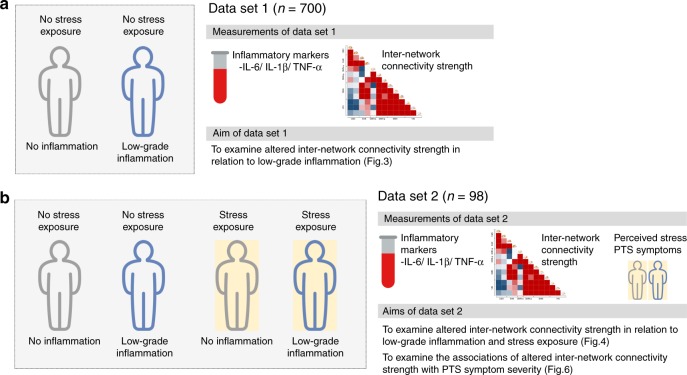
A brief description on the data sets. Two large independent data sets were used in this study. **a** Seven hundred adults in data set 1 were categorized into one of two groups based on serum levels of IL-6, IL-1β, and TNF-α. This was used to determine whether inter-network connectivity strength may be altered in relation to low-grade inflammation. **b** Data set 2 included 52 subjects with repeated exposure to traumatic events and 46 demographically matched healthy individuals without having been exposed to any traumatic event, and were further categorized according to their levels of low-grade inflammation. Alterations in inter-network connectivity strength in relation to both conditions of low-grade inflammation and stress exposure were examined using data set 2 shown in panel **b**. Furthermore, data from the stress-exposed group (*n* = 52) of data set 2 were also used to examine whether alteration in inter-network connectivity strength may contribute to PTS symptom severity. IL-6 interleukin-6, IL-1β interleukin-1β, TNF-α tumor necrosis factor-α, PTS posttraumatic stress.

This study aims to identify altered functional coupling of brain networks that are most relevant to low-grade inflammation and repeated stress exposure, using two independent large-scale data sets comprising healthy individuals and those who had been exposed to repeated trauma. Through the composite scoring of peripheral biomarkers of inflammation, healthy individuals from the first data set (data set 1) (Fig. 1a) are categorized according to low-grade inflammatory and noninflammatory groups, while individuals from the second data set (data set 2) (Fig. 1b) are categorized according to two criteria, including a history of repeated trauma exposure and the composite scoring of peripheral biomarkers of inflammation. Dynamic inter-network correlation matrices between the large-scale brain networks derived from resting-state functional neuroimaging data are used as relevant measures for the brain’s intra- and inter-regional capacity to integrate spatial and temporal information^19^. The functional intra- and inter-regional capacity are measured by the functional connectivity between nodes as derived using two distinct clustering methods in parallel, which are the a priori-defined and data-driven clustering approach, respectively. We also investigate the role of altered functional coupling of the large-scale brain networks related to both low-grade inflammation and repeated stress exposure in subsequent posttraumatic stress (PTS) symptom severity.

## Results

### Sample characteristics and measurements

Two large independent data sets were used to investigate alterations in inter-network connectivity related to low-grade inflammation (data set 1:700 healthy adults categorized into two groups according to their inflammatory activity as measured by the composite scoring of the peripheral biomarkers of inflammation) and combined influence of stress exposure and low-grade inflammation (data set 2:98 adults categorized according to two criteria, including a history of repeated trauma exposure and inflammatory activity) (Fig. 1). Demographic and clinical characteristics of data sets 1 and 2 are shown in Table 1 and Supplementary Table 1.

**Table 1:** Demographic and clinical characteristics of data sets.

Characteristics	Data set 1 (*n* = 700)	Data set 2 (*n* = 98)
Age, mean (SD), y	32.7 (11.6)	33.3 (3.9)
Male, *n* (%)	410 (58.6)	84 (85.7)
Body mass index, mean (SD), kg/m^2^	22.8 (2.6)	23.8 (2.3)
Inflammatory activity		
IL-6, mean (SD), pg/mL	4.70 (3.07)	4.43 (3.12)
IL-1β, mean (SD), pg/mL	2.90 (1.60)	2.21 (1.00)
TNF-α, mean (SD), pg/mL	11.1 (2.5)	11.0 (2.4)
Perceived stress level^a^, mean (SD),	NA	56.4 (25.2)
CAPS^a^, mean (SD), total scores	NA	7.75 (10.55)

*y* year, *SD* standard deviation, *IL-6* interleukin-6, *IL-1β* interleukin-1β, *TNF-α* tumor necrosis factor-α, *CAPS* Clinician-Administered Posttraumatic Stress Disorder Scale for DSM-4, *NA* not applicable.

^a^Perceived stress level was assessed using a visual analog scale to rate the stress level of the stress-exposed participants of data set 2 (*n* = 52) with respect to their worst traumatic experience throughout their career. Posttraumatic stress symptom severity was also measured in the stress-exposed participants of data set 2 using the CAPS (*n* = 52). Source data are provided as a Source Data file.

Serum levels of three pro-inflammatory cytokines were measured as indices of low-grade inflammation: interleukin-6 (IL-6), interleukin-1β (IL-1β), and tumor necrosis factor-α (TNF-α).

**Fig. 2:**
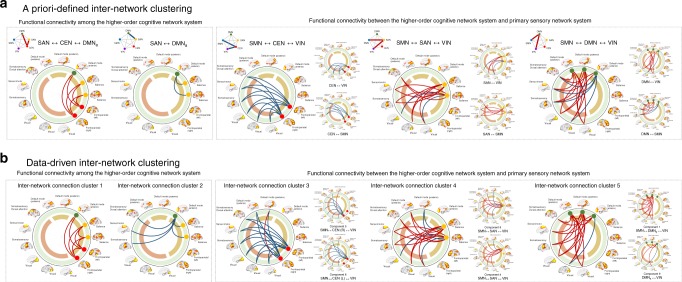
A visual display of inter-network connection clusters selected in this study. **a** Mean connectivity strengths of the positive functional coupling of the SAN–CEN–DMN_a_ and negative functional coupling of SAN–DMN_a_ were selected as the two outcome variables for functional connectivity among the higher-order cognitive network system. Mean connectivity strengths of the SMN–CEN–VIN, SMN–SAN–VIN, and SMN–DMN–VIN were also calculated as the three outcome variables for functional connectivity between the higher-order cognitive network system and primary sensory network system. **b** Five neurobiologically meaningful inter-network connection clusters were selected from a total of 78 inter-network functional connectivity pairs using factor analysis (see also Supplementary Table 2). The two inter-network connection clusters in the left represent functional connectivity among the higher-order cognitive networks, including the CEN, SAN, and DMN_a_. The three inter-network connection clusters on the right indicate functional connectivity between each of the networks belonging to the higher-order cognitive network system and the primary sensory network system, including the DMN and VIN. Red and blue lines indicate the positive and negative coupling of networks, respectively. SAN salience network, DMN default mode network, DMN_a_ default mode network, anterior, CEN central executive network, SMN sensorimotor network, VIN visual network.

We measured the inter-network functional connectivity strength among the higher-order cognitive network system, including the central executive network (CEN), salience network (SAN), and anterior default mode network (DMN_a_), as well as the primary sensory network system, including the sensorimotor network (SMN) and visual network (VIN), to examine the neurobiological correlates of low-grade inflammation and stress exposure (Fig. 2). An a priori-defined inter-network clustering approach was implemented, where the averaged connectivity strengths of the following network node pairs were calculated in each subject and used in subsequent analyses: (1) SAN–CEN–DMN_a_, (2) SAN–DMN_a_, (3) SMN–CEN–VIN, (4) SMN–SAN–VIN, and (5) SMN–DMN–VIN. These networks were selected based on prior knowledge on the specific large-scale functional brain networks that are involved in both low-grade inflammation and stress^13,14^. Correlations between a subset of major representative network nodes that belong to the above-mentioned functional networks were investigated using intra- and inter-network functional connectivity^20,21^.

In addition, a data-driven approach to measuring inter-network functional connectivity strength was also conducted in parallel, using factor analysis based on 78 inter-network functional edges to extract relevant subsets of inter-network connection clusters that revealed common patterns (Fig. 2; Supplementary Table 2). The connection patterns of inter-network clusters 1 through 5 that were derived from the factor analysis were similar to those from the a priori-defined inter-network clustering approach (Fig. 2). Connectivity strengths among the extracted inter-network connection clusters from the data-driven approach were also averaged and included in subsequent analyses.

### Data set 1

**Fig. 3:**
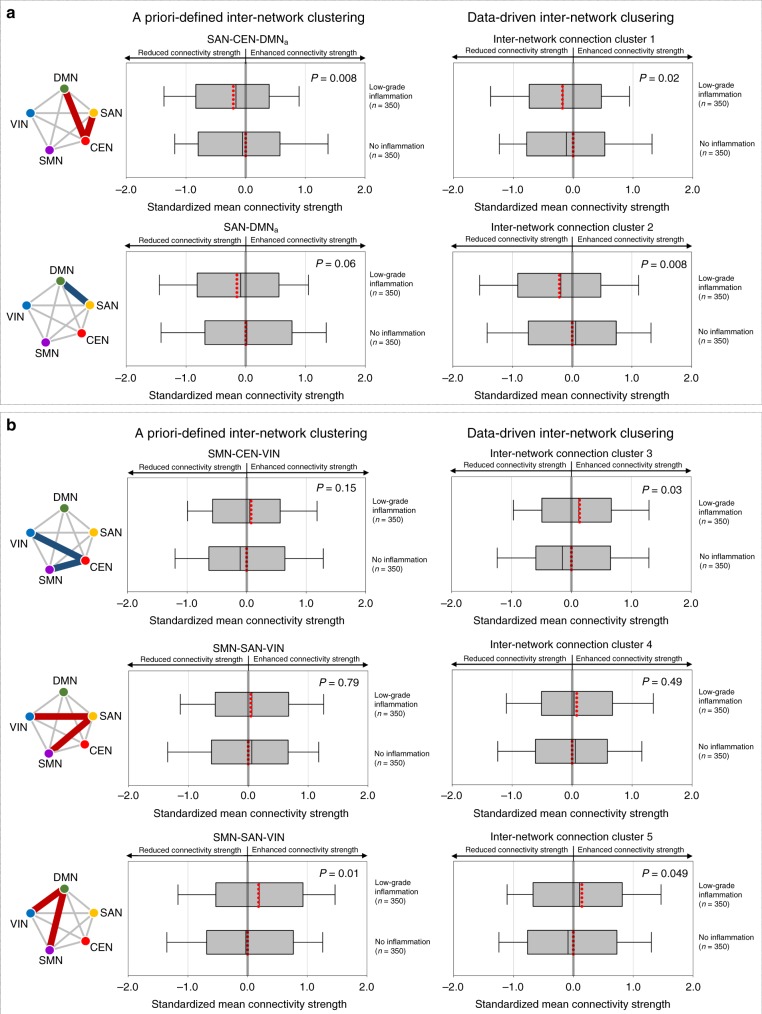
Results from data set 1: altered inter-network connectivity strength in relation to low-grade inflammation. **a** Box-and-whisker plots show the distribution of standardized mean connectivity strength within the higher-order cognitive network system, including the SAN, CEN, and DMN_a_ between the low-grade inflammatory (*n* = 350) and noninflammatory groups (*n* = 350). **b** Box-and-whisker plots show the distribution of standardized mean connectivity strength between the higher-order cognitive network system and the primary sensory network system between the low-grade inflammatory (*n* = 350) and noninflammatory groups (*n* = 350). Box-and-whisker plots represent the mean values (red dotted lines inside boxes), median values (black lines inside the boxes), the inter-quartile range (bottom and top ends of the boxes), and the 10th and 90th percentiles of the data (whiskers). Red and blue lines in the schematic diagram for inter-network connection clusters indicate the positive and negative coupling of networks, respectively. Group differences were calculated using the generalized linear models after adjusting for age and sex. *P* values (for two-sided testing) in the graphs are permutation-adjusted *P* values (10,000 permutations). Source data are provided as a Source Data file. SAN salience network, DMN default mode network, DMN_a_ default mode network, anterior, CEN central executive network, SMN sensorimotor network, VIN visual network.

To elucidate the changes in inter-network connectivity strength related to low-grade inflammation, the standardized mean connectivity strengths of the inter-network connection clusters were compared between the low-grade inflammatory (*n* = 350) and noninflammatory (*n* = 350) groups of data set 1. For the inter-network connection clusters of the higher-order cognitive network system, the standardized mean connectivity strengths of the positive coupling among the SAN–CEN–DMN_a_ (generalized linear model [GLM], *z* = −2.66, permutation-adjusted *P* = 0.008) were significantly reduced in the low-grade inflammatory group relative to the noninflammatory group. A similar pattern of reduced mean connectivity strengths of inter-network cluster 1 derived from the data-driven approach was observed in relation to low-grade inflammation (GLM, *z* = −2.31, permutation-adjusted *P* = 0.02) (Fig. 3a; Supplementary Table 3). Furthermore, although the difference did not reach statistical significance, mean connectivity strength of the negative functional coupling between the SAN and DMN_a_ was reduced (GLM, *z* = −1.89, permutation-adjusted *P* = 0.06). Similarly, mean connectivity strengths of the inter-network cluster 2 that was mainly involved in the connections between the SAN and DMN_a_ were significantly reduced in the low-grade inflammatory group relative to the noninflammatory group (GLM, *z* = −2.72, permutation-adjusted *P* = 0.008) (Fig. 3a; Supplementary Table 3).

For the connections between the higher-order cognitive network system and primary sensory network system, there was a significant difference in the standardized mean connectivity strength of the SMN–DMN–VIN between the two groups (GLM, *z* = 2.48, permutation-adjusted *P* = 0.01), while no between-group differences in the standardized mean connectivity strengths were found in SMN–CEN–VIN as well as SMN–SAN–VIN connection clusters (Fig. 3b; Supplementary Table 3). A similar pattern of between-group difference was observed in the data-driven clustering approach for inter-network clusters 3, 4, and 5 (Fig. 3b; Supplementary Table 3).

To ensure the robustness of the results, we have repeated the analyses from above while including potential confounding factors as additional covariates. Similar results were obtained when the amount of head motion as measured by framewise displacement (FD) and body mass index (BMI) was included as an additional covariate into the statistical model, respectively. Details on the statistical values for these analyses are described in Supplementary Note 1.

### Data set 2

**Fig. 4:**
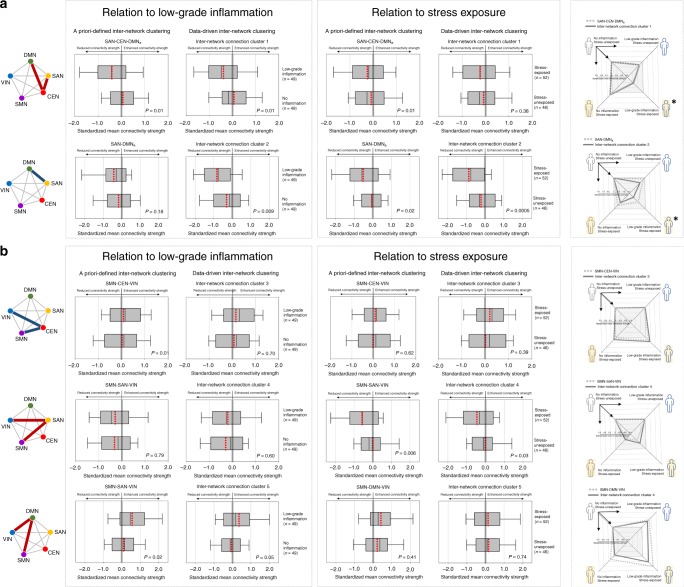
Results from data set 2: altered inter-network connectivity strength in relation to low-grade inflammation and stress exposure. **a** Box-and-whisker plots show the distribution of standardized mean connectivity strength within the higher-order cognitive network system, including the SAN, CEN, and DMN_a_ between the low-grade inflammatory (*n* = 49) and the noninflammatory groups (*n* = 49) (left box), as well as the stress-exposed (*n* = 52) and -unexposed groups (*n* = 46) (right box). **b** Box-and-whisker plots show the distribution of standardized mean connectivity strength for the higher-order cognitive network system and the primary sensory network system between the low-grade inflammatory (*n* = 49) and the noninflammatory groups (*n* = 49) (left box), as well as the stress-exposed (*n* = 52) and -unexposed groups (*n* = 46) (right box). Box-and-whisker plots represent the mean values (red dotted lines inside boxes), median values (black lines inside the boxes), the inter-quartile range (bottom and top ends of the boxes), and the 10th and 90th percentiles of the data (whiskers). Red and blue lines in the schematic diagram for inter-network connection clusters indicate the positive and negative coupling of networks, respectively. Group differences were calculated using the generalized linear models after adjusting for age and sex. *P* values (for two-sided testing) in the bar graph are permutation-adjusted *P* values (10,000 permutations). Radar graphs in panels **a** and **b** demonstrate comparisons of inter-network connectivity strength between the noninflammatory/stress-unexposed group (*n* = 24) and each of the remaining three groups, including the low-grade inflammatory/stress-unexposed (*n* = 22), noninflammatory/stress-exposed (*n* = 25), and low-grade inflammatory/stress-exposed group (*n* = 27), respectively. Asterisks indicate a statistically significant between-group difference in mean connectivity strengths obtained by both the a priori-defined and data-driven inter-network clustering approaches. Source data are provided as a Source Data file. SAN salience network, DMN default mode network, DMNa default mode network, anterior, CEN central executive network, SMN sensorimotor network, VIN visual network.

Findings from the comparisons of inter-network functional connectivity between the low-grade inflammatory (*n* = 49) and noninflammatory (*n* = 49) groups of data set 2 were similar to those observed in data set 1. Specifically, the standardized mean connectivity strengths among the higher-order cognitive network system were reduced in the low-grade inflammatory group as compared with the noninflammatory group (a priori-defined inter-network clustering: SAN–CEN–DMN_a_, GLM, *z* = −2.53, permutation-adjusted *P* = 0.01; SAN–DMN_a_, GLM, *z* = −1.35, permutation-adjusted *P* = 0.18) (data-driven inter-network clustering: inter-network cluster 1, GLM, *z* = −2.48, permutation-adjusted *P* = 0.01; inter-network cluster 2, GLM, *z* = −2.61, permutation-adjusted *P* = 0.009) (Fig. 4a; Supplementary Table 4). Altered inter-network functional connectivity in relation to stress exposure was also examined by comparing the standardized mean connectivity strengths of inter-network connection clusters between the stress-exposed (*n* = 52) and -unexposed (*n* = 46) groups. The stress-exposed group had reduced connectivity strengths in the SAN–DMN_a_ (GLM, *z* = −2.41, permutation-adjusted *P* = 0.02) as well as SMN–SAN–VIN (GLM, *z* = −2.84, permutation-adjusted *P* = 0.006), as compared with the stress-unexposed group (Fig. 4). A similar pattern of between-group differences was observed in the respective inter-network clusters from the data-driven inter-network clustering approach (inter-network cluster 2, GLM, *z* = 3.38, permutation-adjusted *P* = 0.0005; inter-network cluster 4, GLM, *z* = −2.20, permutation-adjusted *P* = 0.03) (Fig. 4). Furthermore, we explored whether such alterations in inter-network connectivity strength related to stress exposure occur regardless of low-grade inflammation. In these exploratory analyses, differences in connectivity strength for the functional coupling of the SAN–DMN_a_ (GLM, *z* = −2.46, permutation-adjusted *P* = 0.01) as well as SMN–SAN–VIN (GLM, *z* = −2.83, permutation-adjusted *P* = 0.007) between the stress-exposed and -unexposed groups remained statistically significant even after adjusting for inflammatory activity (data-driven inter-network clustering: inter-network cluster 2, GLM, *z* = −3.57, permutation-adjusted *P* = 0.0004; inter-network cluster 4, GLM, *z* = −2.19, permutation-adjusted *P* = 0.03). There were no other between-group differences in connectivity strength according to stress exposure for the remaining inter-network connection clusters as acquired by a priori-defined as well as data-driven approaches (Fig. 4; Supplementary Table 4). The results remained unchanged with FD and BMI included as additional covariates, respectively (Supplementary Note 1).

Post hoc analyses demonstrated significant reductions in inter-network connectivity strength among the higher-order cognitive network system in the low-grade inflammatory/stress-exposed group as compared with the noninflammatory/stress-unexposed group (a priori-defined inter-network clustering: SAN–CEN–DMN_a_, GLM, *z* = −2.17, permutation-adjusted *P* = 0.02; SAN–DMN_a_, GLM, *z* = −2.39, permutation-adjusted *P* = 0.01) (data-driven inter-network clustering: inter-network cluster 1, GLM, *z* = −2.19, permutation-adjusted *P* = 0.03; inter-network cluster 2, GLM, *z* = −4.01, permutation-adjusted *P* = 0.0001) (radar charts of Fig. 4a).

**Fig. 5:**
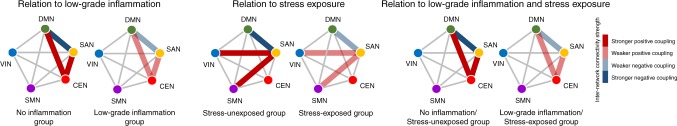
Schematic representation of the alterations in inter-network connectivity strength in relation to low-grade inflammation (left), stress exposure (middle), and the presence of both conditions (right). Red and blue lines of the schematic diagram for inter-network clusters indicate the positive and negative coupling of networks, respectively. DMN default mode network, SAN salience network, CEN central executive network, SMN sensorimotor network, VIN visual network.

A schematic representation of altered inter-network connection clusters related to low-grade inflammation, stress exposure, and both conditions, respectively, is shown in Fig. 5.

**Fig. 6:**
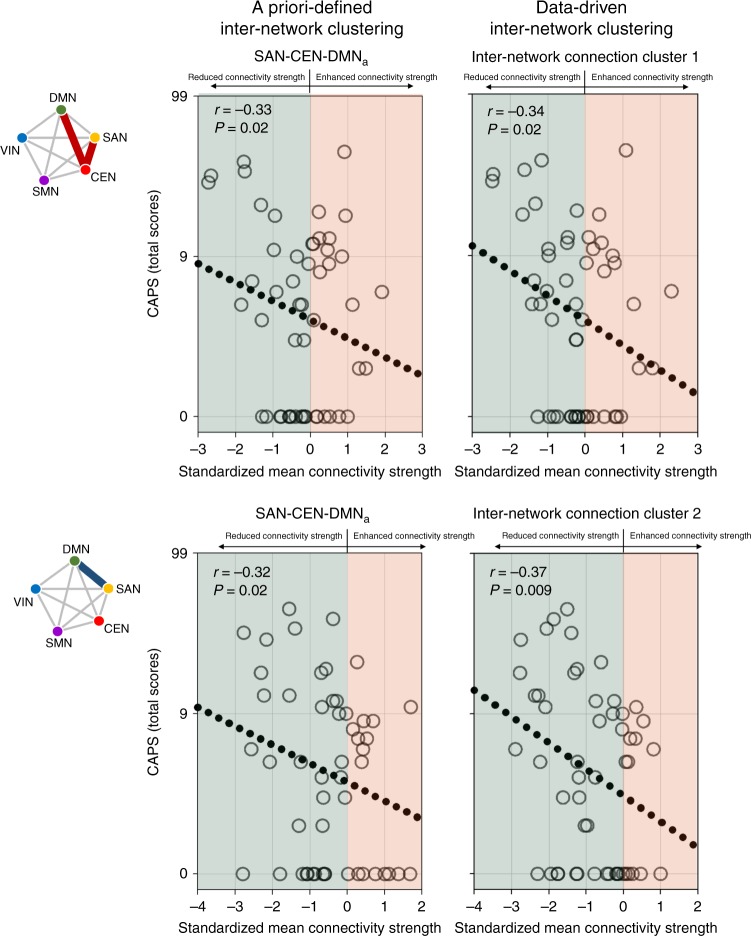
Associations between altered inter-network connectivity strength and posttraumatic stress (PTS) symptom severity. Greater functional coupling among the SAN–CEN–DMN_a_ (*r* = −0.33, *P* = 0.02) or inter-network connection cluster 1 (*r* = −0.34, *P* = 0.02) was associated with lower PTS symptom severity. Reduced negative coupling between the SAN and DMN_a_ (*r* = −0.32, *P* = 0.02) or inter-network connection cluster 2 (*r* = −0.37, *P* = 0.009) was associated with higher PTS symptom severity. Partial correlation analyses were used to assess the relationships between altered inter-network connectivity strength and PTS symptom severity in the stress-exposed group (*n* = 52) of data set 2, including age and sex as covariates. Red and blue lines in the schematic diagram for inter-network clusters indicate the positive and negative coupling of networks, respectively. Source data are provided as a Source Data file. CAPS Clinician-Administered Posttraumatic Stress Disorder Scale for DSM-4, DMN default mode network, DMN_a_ default mode network, anterior, SAN salience network, CEN central executive network, SMN sensorimotor network, VIN visual network.

In the stress-exposed group of data set 2 (*n* = 52), associations between PTS symptom severity as measured using the Clinician-Administered Posttraumatic Stress Disorder Scale for DSM-4 (CAPS)^22^ and altered inter-network connectivity strength in relation to low-grade inflammation and stress exposure were examined. Reduced connectivity strength in the functional coupling of the SAN–CEN–DMN_a_ (partial correlation analysis, *r* = −0.33, *P* = 0.02) as well as SAN–DMN_a_ (partial correlation analysis, *r* = −0.32, *P* = 0.02) was significantly associated with higher PTS symptom severity, respectively (Fig. 6). A similar pattern was observed in the data-driven approach in terms of the relationships between PTS symptom severity and connectivity strength of inter-network clusters 1 (partial correlation analysis, *r* = −0.34, *P* = 0.02) and 2 (partial correlation analysis, *r* = −0.37, *P* = 0.009), respectively. Associations between PTS symptom severity and connectivity strength of the other inter-network connection clusters identified in this study are described in Supplementary Table 5. As exploratory analyses, the relationships between inter-network connectivity strength and each of the CAPS subscale scores for re-experiencing symptoms, avoidance symptoms, and hyperarousal symptoms were also examined and described in Supplementary Table 6.

## Discussion

This study aimed to elucidate the shared large-scale brain network correlates in low-grade inflammation and stress exposure. Our most prominent finding is that inter-network functional connectivity among the SAN, DMN_a_, and CEN is particularly vulnerable to both inflammation and stress. Furthermore, altered functional connectivity of these networks was associated with subsequent PTS symptom severity in individuals with repeated stress exposure. The current findings strongly suggest that the combined influences of low-grade inflammation and stress exposure may increase the risk of PTSD through disruption in the functional coupling of the higher-order cognitive network systems such as the SAN, DMN_a_, and CEN^15,16^.

To date, a strong correspondence between low-grade inflammation and increased risk of various psychiatric disorders has been supported by epidemiological and animal studies^4,6,11^. The strength of the current study is the investigation of its neural network mechanisms, such that the potential underlying pathway between low-grade inflammation and its associations with clinical outcome may be elucidated in the case of high-risk individuals with repeated stress exposure.

Consistent with previous studies on the pathological inflammatory conditions such as rheumatoid arthritis as well as experimental conditions of receiving immunogenic substances^23–25^, we found that functional connectivity among networks that subserve the regulation of emotion and cognition^15,16^ is significantly influenced by low-grade inflammation. In addition to this supportive finding, we also demonstrated that functional coupling of the large-scale brain networks that are closely associated with stress overlaps with those related to low-grade inflammation. The higher-order cognitive network systems identified in the current study, including the SAN, CEN, and DMN_a_, have been previously suggested to take part in the pathology of PTSD^26–28^. As such, the current findings suggest that the influence of low-grade inflammation on the functional connectivity of these brain networks may be potentiated by ongoing stress exposure. In other words, the presence of low-grade inflammation in individuals with repeated stress exposure may indicate a greater risk of PTSD.

The triple-network model involves the modulatory role of the SAN in the switching between the DMN and CEN according to the presence or absence of a task, which highlights the dynamic interactions of these three networks^28,29^. Although previous findings were inconsistent in the directionality among these three networks, the SAN has shown to be consistently altered in relation to peripheral inflammation^12,24,30,31^. Current findings on aberrant functional coupling of these three networks suggest the significant roles of the triple networks in the combined influence of inflammation and stress on subsequent PTS symptom severity. In addition, the dysfunctional interactions between the SAN and DMN may contribute to the hypervigilant or hyperarousal symptoms often found in PTSD, potentially by inappropriate responsiveness to external stimuli^26,27,32,33^. Our exploratory findings regarding the significant association between hyperarousal symptom severity and altered functional connectivity among the SAN, DMN_a_, and CEN may support this speculation (Supplementary Table 6).

Aberrant functional organization of any part of the three networks within the triple-network model may also affect other remaining networks, resulting in the clinical manifestation of certain psychiatric disorders^29^. Considering this, alterations in inter-network functional connectivity induced by low-grade inflammation as found in this study may play additional or even synergic roles in the regulation of emotion in the case of repeated stress exposure.

Our study also found that stress exposure may influence functional coupling of the SAN to the DMN_a_ as well as to the SMN and VIN. This is consistent with the previous finding which reported that psychosocial stress may alter functional connectivity of the insula and anterior cingulate, both of which were associated with increased levels of IL-6 and TNF-α^30^. Considering that IL-6 has shown to associate with the functional connectivity within the DMN^34^, the current findings may further support that the connectivity among the triple-network model may act as neural correlates of PTS symptoms in combination with low-grade inflammation. In addition, growing evidence suggests that the insular cortex, a key node of the SAN, and its functional link to other brain regions play a crucial role in the central representation of inflammation^35–37^ as well as anxiety or depression^36,38^. Specifically, our findings of reduced functional connectivity regarding the SAN that are also associated with greater PTS symptom severity may be consistent with previous studies that have noted decreased SAN connectivity following induced transient systemic inflammation^39^, as well as the interaction effect between functional connectivity of the brain with clinical symptom severity^18^.

In this study, we repeated our analyses with inter-network connection matrices using various dimensionalities, which may allow to resolve for the tendency of overfitting the data as well as provide a much finer analysis that may mimic the alternative approach of region-based high-resolution parcellation. It is noteworthy that the main findings of the current study were consistent and supported across various dimensionalities (Supplementary Note 2). Therefore, this study may provide supportive evidence for the reliability of the findings as well as the replication of previous studies, which noted the consistent decomposition using independent component analysis (ICA) across a range of dimensionality^40,41^.

In order to provide both intuitive and integrative approaches to the clustering of brain network couplings, this study grouped the functional connections among 13 network nodes into five inter-network clusters by implementing both a priori-defined and data-driven inter-network clustering methodologies, respectively. Although minor differences were found in the composition of individual functional connections, the results regarding alterations in inter-network connectivity strength according to low-grade inflammation and stress exposure were substantially similar between clusters that were derived from the two distinct clustering approaches.

The following limitations should be considered. In this study, the stress-exposed group consisted of firefighters, and therefore may not be representative of all types of stress-exposed populations. Although the stress-exposed group in the current study experienced one or more traumatic events and is considered to be at high risk for PTSD^42,43^, future studies should be more inclusive in their definition of psychosocial stress. Also, this study did not include individuals with transient surges in inflammation, such as infection, or those with chronic inflammatory conditions, including rheumatoid arthritis in investigating the effects of inflammation. In addition, inflammatory activity was analyzed in a general population using a median split. While the current sample may reflect the real-life influence of the interaction between low-grade inflammation and stress, future studies that include the vast spectrum of inflammation are warranted. Furthermore, the current study found that inter-network connectivity of the SAN may alter in relation to stress exposure, as demonstrated in data set 2. Although these findings remained significant after the adjustment of inflammatory activity, future studies with a larger sample would be necessary to identify the specific inter-network connectivity that may be prone to stress exposure, regardless of the subjects’ inflammatory activity.

This study also used a multiplex-based immunoassay to measure pro-inflammatory cytokines as indicators of low-grade inflammation. Multiplex-based immunoassay may have an advantage to perform large-scale epidemiological studies, as it enables simultaneous quantification of several different cytokines at relatively small sample volumes^44,45^. However, the results obtained using multiplex-based immunoassay are to be cautiously interpreted in the context of multiplex technologies^45,46^. Reliability and reproducibility are particularly important factors to consider when using multiplex-based methodologies^45,47^. In this study, intra- and inter-assay coefficients of variation (CVs) for the pro-inflammatory cytokine measurements exhibited patterns of consistency, which may support that the current methodologies yield acceptable performance. In addition, the status of low-grade inflammation was defined based on composite scoring of the standardized and averaged values of three pro-inflammatory cytokines IL-6, IL-1β, and TNF-α, instead of using the raw values of each individual cytokine. This approach may allow minimization of potential data noise due to measurement error or missing data^48^. Despite these efforts to increase performance and validity of pro-inflammatory cytokine measurements, future studies using a more conventional method such as the enzyme-linked immunosorbent assay should be performed to replicate the current findings.

Furthermore, participants of data set 1 had not experienced any type of serious traumatic event, and therefore perceived stress level toward an index trauma was not assessed. However, considering the growing evidence that indicates a significant association between stress and inflammation^5–8^, future studies that assess the potential effects of perceived stress levels from daily-life events are warranted to provide further details regarding the interactive influence between low-grade inflammation and stress exposure.

Previous studies reported the potential link between exposure to persistent or repetitive psychosocial stress and low-grade inflammation and vice versa^8,11^. While our study did not find differences in inflammatory activity between the stress-exposed and -unexposed groups (Supplementary Table 1), possible bidirectional influences of low-grade inflammation and stress exposure should be considered in interpreting the results from our cross-sectional study. Furthermore, the current cross-sectional study design may not distinguish whether low-grade inflammation of the study participants was episodic or sustained, and the directionality between inflammation, network alterations, and clinical manifestations could not be determined.

In summary, our study provides evidence for the potential brain mechanisms underlying low-grade inflammation and stress in subsequent manifestations of PTSD. Our findings indicate that increased inflammatory activity may render individuals who are exposed to trauma at an even higher risk of PTSD. The current results provide a proof of concept for the potential use of anti-inflammatory treatment methods in the prevention or treatment for PTSD for individuals who are repeatedly exposed to stress.

## Methods

### Data sets

All analyses were performed in two large independent data sets of South Korea. Data set 1 (*n* = 700) was used to investigate alterations in inter-network functional connectivity in relation to low-grade inflammation. Study participants in data set 1 were healthy adult volunteers (mean [standard deviation, SD], 32.7 [11.6] years; 410 men and 290 women) recruited through community advertisement. The exclusion criteria were (1) significant medical, neurological, or psychiatric disorders as assessed using the medical, neurological, and psychiatric history taking, including the assessment through the Structured Clinical Interview for DSM-4 (SCID)^49^ and routine laboratory tests, (2) significant structural abnormalities as found in the brain magnetic resonance imaging (MRI) scans confirmed by an experienced neuroradiologist, (3) having experienced any type of serious traumatic event, (4) a history of severe traumatic brain injury, or (5) any contraindications to MRI.

Data set 2 (*n* = 98) included 52 firefighters who have been repeatedly exposed to direct and/or indirect forms of life-threatening traumatic events (the stress-exposed group, mean [SD], 33.6 [4.0] years; 45 men and 7 women) and 46 healthy individuals who were not exposed to any significant traumatic event (the stress-unexposed group, mean [SD], 33.0 [3.8] years; 39 men and 7 women) to investigate alterations in inter-network functional connectivity related to stress exposure. All firefighters in the stress-exposed group were employed at fire stations within the Seoul metropolitan area of South Korea, while the demographically matched healthy individuals in the stress-unexposed group were recruited through community advertisement in the Seoul metropolitan area. Participants of data set 2 were assessed using comprehensive history taking for the screening of any significant medical, neurological, or psychiatric disorders as well as the SCID. Individuals exposed to trauma in data set 2 had met the guidelines of Criterion A of the CAPS, which is defined as having experienced one or more index traumatic event. Individuals exposed to trauma were also assessed using the CAPS as to determine the diagnosis of PTSD and the severity of PTS symptoms, along with perceived stress level. Among the individuals who were assessed for trauma exposure, those who met the diagnostic criteria for PTSD were excluded from the study, and only those with subclinical levels of PTS symptoms were included as part of the stress-exposed group for further analysis. Other than the exclusion criterion regarding trauma exposure, the inclusion and exclusion criteria applied to individuals in data set 2 were identical to those of data set 1.

All participants from data sets 1 and 2 underwent MRI scans at Ewha Brain Institute of Ewha W. University, Seoul, South Korea, and were assessed for serum levels of pro-inflammatory cytokines. Demographic and clinical characteristics of both data sets are described in Table 1 and Supplementary Table 1. A description on data sets and measurements is also depicted in Fig. 1.

The Institutional Review Board of Ewha W. University approved the study protocol, and all participants provided written informed consent. All of the study processes were in alignment with the Declaration of Helsinki as well as national and institutional regulations and guidelines.

### Measurement of inflammatory activity

Serum levels of three pro-inflammatory cytokines were measured as indices of low-grade inflammation: IL-6, IL-1β, and TNF-α. A composite value for inflammatory activity was calculated by averaging the standardized *z* scores of the three cytokine measures^50,51^. For the current composite scoring methods, each of the three cytokine measures for low-grade inflammation was computed according to sex to consider the sex-specific differences in cytokine values^52,53^. For instance, standardized *z* scores for all cytokine measures were calculated in each sex group using the mean and standard deviation for the relevant sex group. Study participants from data set 1 were categorized into either of two groups based on the median inflammatory composite value: the low-grade inflammatory (*n* = 350) vs. noninflammatory (*n* = 350) groups. A similar process of categorization was applied to data set 2 that yielded the low-grade inflammatory (*n* = 49) vs. noninflammatory (*n* = 49) groups.

Serum levels of IL-6, IL-1β, and TNF-α were measured for both data sets 1 and 2 according to the following procedure. Peripheral blood samples were drawn from the participants and collected in a serum-separating tube in the morning between 9 AM and 11 AM. The sample was reserved for 30 min in room temperature, then centrifuged at 3000 rpm to separate the serum. Aliquots of the serum were stored immediately after the centrifugation at a temperature of −70 °C until the time of analyses. Each of the serum levels of IL-6, IL-1β, and TNF-α was assessed per sample in duplicate and quantified using a multiplexed, fluorescent bead-based immunoassay (MILLIPLEX^Ⓡ^ MAP Human High Sensitivity T Cell Magnetic Bead Panel, Merck Millipore, MA, USA) on a Luminex xMAP® platform (Luminex Corporation, TX, USA)^54^. Assays were performed according to the manufacturer’s instructions. Specifically, 25 μL of serum was incubated with antibody-coupled beads. Following a series of washes, 50 μL of a biotinylated detection antibody and 50 μL of streptavidin–phycoerythrin were added to the beads in order to detect the reaction mixture. The bead sets were analyzed using a flow-based Luminex™ 200 system (Luminex Corporation, TX, USA). The mean fluorescence intensity of the raw data was captured using the Luminex xPONENT software (Luminex Corporation, TX, USA). The concentrations of the cytokines in each sample were calculated using the MILLIPLEX-Analyst (Viagene Tech, MA, USA) with a five-parameter logistic regression to compute sample concentrations from the standard curves. For all samples, the mean values of the duplicate were reported, and the intra-assay CVs were calculated as 12.6%, 11.8%, and 8.4% for IL-6, IL-1β, and TNF-α, respectively. Inter-assay CVs, using a subset of samples repeatedly assessed, were also within the acceptable range (19.0%, 15.1%, and 12.4% for IL-6, IL-1β, and TNF-α, respectively). The detection limits for the assays of IL-6, IL-1β, and TNF-α were 0.18–750 pg/ml, 0.49–2000 pg/ml, and 0.45–1750 pg/ml, respectively, as according to the manufacturer’s instructions. There were no undetectable values in the samples of TNF-α. In contrast, seven and six samples of IL-6 and IL-1β were below the lower limit of detection, respectively. Measurements below the lower limit of detection for IL-6 and IL-1β were recorded as one-half of the lower limit of detection. The range of serum concentrations for IL-6^55–57^, IL-1β^55,56^, and TNF-α^55–57^ obtained in this study using a multiplexed, fluorescent bead-based immunoassay were comparable with those reported in previous literature on healthy individuals using similar methodologies.

All the measurements of inflammatory activity were performed by personnel who were blind to the participants’ information at the Green Cross Laboratories (Yongin, South Korea), certified by the College of American Pathologists’ Laboratory Accreditation Program and the German External Quality Assessment Scheme.

### MRI data acquisition

High-resolution structural images were acquired using a three-dimensional T1-weighted magnetization-prepared rapid gradient echo imaging sequence with the following parameters: echo time, 3.4 ms; repetition time, 7.4 ms; flip angle, 8°; field of view, 220 × 220 mm^2^; slice thickness, 1 mm; 180 contiguous sagittal slices. Resting-state functional MRI (fMRI) scans were performed using an echo planar imaging sequence with the following acquisition parameters: echo time, 21 ms; repetition time, 2000 ms; flip angle, 76°; field of view, 220 × 220 mm^2^; slice thickness, 3.5 mm, 200 volumes, 38 slices. To reduce motion artifact, the dynamic stabilization option was additionally employed^58^. During resting-state fMRI data acquisition, participants were asked to stay awake while keeping their eyes closed and let their minds wander freely. Fluid-attenuated inversion recovery axial images were also acquired to screen for any gross neuroradiological abnormalities, using the following parameters: echo time, 276 ms; repetition time, 4800 ms; inversion time = 1650 ms; field of view, 240 × 240 mm^2^; slice thickness, 0.56 mm.

### Preprocessing of resting-state fMRI data

Head movement was inspected through the calculation of the mean FD for each resting-state fMRI image^59^. Subjects with qualified fMRI images where the mean FD was <0.3 mm were included in the study. The distribution of FD for participants in data sets 1 and 2 is presented in Supplementary Fig. 1. In both data sets 1 and 2, the amount of head motion fell within the 0.3-mm threshold, where the mean FD values of 90% of the individual images were 0.13 mm and 0.16 mm for data sets 1 and 2, respectively.

Resting-state fMRI data were preprocessed using the FMRIB Software Library tools (FSL, http://www.fmrib.ox.ac.uk/fsl). The standard preprocessing steps were as follows: rigid body coregistration for head motion correction^60^; brain extraction using the FSL Brain Extraction Tool; spatial smoothing (Gaussian kernel of full width at half maximum, 5.0 mm); high-pass temporal filtering, 0.01 Hz. Resting-state fMRI data image of each individual was first co-registered to the corresponding high-resolution T1-weighted image. These co-registered images were linearly registered to the standard Montreal Neurological Institute (MNI) 152 template with 12 degrees of freedom, subsequently resampled to 4-mm isotropic voxel space. In addition, a data-driven denoising strategy was employed to remove head motion and structural artifacts at an individual image level using single-subject ICA implemented by Multivariate Exploratory Linear Optimized Decomposition into Independent Components (MELODIC) followed by FMRIB’s ICA-based Xnoiseifier (FIX)^61,62^.

### Group ICA

In order to generate a set of group-averaged network nodes, a model-free and data-driven approach of group ICA with a dual-regression algorithm was applied to decompose the preprocessed four-dimensional fMRI images of all 798 individuals from data sets 1 and 2 into a set of three-dimensional spatial maps and one-dimensional time series^41,63^. The three-dimensional spatial maps were analyzed using a temporal concatenation approach with a predetermined dimensionality of 25. Resting-state networks (RSNs) were identified by visual inspection of the group-independent components, where components were labeled as either RSNs or artifacts based on a guideline from previous literature^41^. A total of 13 RSNs were selected and thresholded at z = 3 (*P* = 0.001) and used as network nodes for constructing the inter-network connection matrix: (1) right frontoparietal network; (2) left frontoparietal network; (3) SAN 1; (4) SAN 2; (5) DMN_a_; (6) DMN, posterior (DMN_p_)1; (7) DMN_p_ 2; (8) somatosensory network 1; (9) SMN; (10) somatosensory network 2; (11) VIN 1; (12) VIN 2; (13) VIN 3 (Supplementary Fig. 2). These network nodes were further grouped into five categories that are the CEN (red circles in Supplementary Fig. 2a), SAN (yellow circles in Supplementary Fig. 2a), DMN (green circles in Supplementary Fig. 2a), SMN (blue circles in Supplementary Fig. 2a), and VIN (purple circles in Supplementary Fig. 2a).

Group ICAs were also performed on all 798 individuals with higher dimensionalities to demonstrate the consistency of inter-network functional connectivity patterns across a range of dimensionality^20,21,40^, with various dimensionalities of 25, 77 (automatic estimated), and 200 (Supplementary Note 2). The automatic estimation of optimal dimensionality by Bayesian dimensionality estimation technique^64^ was implemented and 77 components were finally determined. In addition, a predetermined dimensionality of 200 was also applied to this exploratory analysis. After the group ICAs with 77 and 200 dimensionalities, respectively, brain network components that did not fall under the interests of the current study aims, such as the cerebellum, subcortex, and temporal networks as well as noise components were removed. Finally, 29 and 78 network components were identified as candidate network nodes from the group ICA with 77 and 200 dimensionalities, respectively, for the construction of inter-network connection matrices (Supplementary Figures 3 and 4). Similar to the 13 × 13 matrix with a dimensionality of 25, candidate network nodes in each matrix with those of higher dimensionalities were further classified into one of major representative networks, including the higher-order cognitive network system of the CEN, SAN, and DMN_a_, as well as the primary sensory network systems, including the SMN and VIN.

### Construction of the inter-network matrix for each subject

We measured the strength of inter-network functional edges to examine the neurobiological correlates of low-grade inflammation and stress exposure. The temporal correlation coefficient between each pair of the candidate network nodes was computed based on individual time series using FSLNets (http://fsl.fmrib.ox.ac.uk/fsl/fslwiki/FSLNets). These correlation coefficients were Fisher z-transformed and extracted for each subject, which were defined as the inter-network connectivity values. A 13 × 13 correlation matrix was then generated for each subject as to represent correlations between all pairs of network nodes^20^. A lower absolute inter-network connectivity value within the matrix reflects reduced inter-network connectivity, and the sign indicates positive or negative functional coupling of networks. For a clear interpretation of the results, these connectivity values were transformed into inter-a.

### Selection of inter-network clusters for outcome variables

Among 78 inter-network functional edges, the following inter-network edges were selected a priori, where the connectivity strength of each selected edge was averaged as the outcome variable for functional connectivity among the higher-order cognitive network system (positive coupling of SAN–CEN–DMN_a_; negative coupling of SAN–DMN_a_) and functional connectivity between higher-order cognitive network system and primary sensory network system (SMN–CEN–VIN; SMN–SAN–VIN; SMN–DMN–VIN).

In order to minimize arbitrariness in the grouping of brain regions, we also implemented an intuitive approach to clustering functional edges by conducting a factor analysis. Specifically, a principal component analysis with varimax rotation was conducted based on all possible 78 inter-network edges of a 13 × 13 inter-network connection matrix to reduce the number of inter-network edges into more meaningful and distinct clusters with an eigenvalue of 1.5 and at least 2% of variance. With this data-driven inter-network clustering approach, 11 components that are interdependent as well as neurobiologically relevant were finally identified (Supplementary Table 2). The identified 11 components explain 65.5% of the total proportion of variance (Supplementary Fig. 5). Among these, components 10 and 11 that included functional connections between network nodes belonging to the CEN and those belonging to the DMN_a_ or SAN were defined as inter-network connection cluster 1. Inter-network connection cluster 1 also included functional connections among network nodes of the CEN as well as those among network nodes of the SAN. Component 2 that included functional connections between the DMN_a_ and SAN, as well as DMN_a_ and SMN, was defined as inter-network connection cluster 2. Although the functional connectivity pattern of inter-network cluster 2 was similar to that of the a priori-defined SAN–DMN_a_, inter-network cluster 2 additionally included the negative coupling of the DMN_a_ and SMN. Component 5 included functional connections of the right frontoparietal network node with three network nodes belonging to the SMN, 3 network nodes belonging to the VIN, and 2 network nodes belonging to the DMN_p_, while component 6 consisted of functional connections between the left frontoparietal network node and network nodes from the SMN, VIN, and DMN_p_. Components 5 and 6 that represented the functional connections of the CEN with SMN, VIN, and DMN_p_, were defined as inter-network connection cluster 3. Component 4 consisted of functional connections between the SAN2 network node and network nodes from the SMN, VIN, and DMN_p_. Functional connections between the SAN1 network node and network nodes from the SMN, VIN, and DMN_p_ were classified as component 8. We defined components 4 and 8 that represented functional connections of the SAN with the SMN, VIN, and DMN_p_ as inter-network connection cluster 4. Component 1 included functional connections of network nodes belonging to the DMN_p_ and those of the SMN and VIN, while component 9 consisted of functional connections of DMN_a_ with network nodes belonging to the DMN_p_ and those belonging to the VIN. We defined components 1 and 9 as inter-network connection cluster 5.

The pattern and individual functional connections belonging to the inter-network connection clusters that were defined using a priori-defined and data-driven inter-network clustering approaches are presented in Fig. 2a, b, respectively.

An averaged inter-network connectivity strength of all pairs of connections for each inter-network connection cluster was standardized using the mean value of the reference group and pooled SD. Finally, the standardized mean connectivity strength of each inter-network connection cluster was used for subsequent analysis.

In addition, using the group ICA with a higher dimensionality of 77 and 200, the final 29 × 29 and 78 × 78 correlation matrices, respectively, were generated where the measures for inter-network connectivity values (Supplementary Fig. 6) and inter-network connectivity strength were calculated at an inter-network edge level. The averaged connectivity strength was also calculated by averaging the connectivity strength of all pairs of edges among the major representative networks (CEN, SAN, DMN, SMN, and VIN) for further exploratory analyses. Details of the exploratory analyses on inter-network connection matrices with a higher resolution of parcellation are presented in Supplementary Note 2, Supplementary Table 7, and Supplementary Figs. 7 and 8.

### Perceived stress levels and PTS symptom severity assessment

For individuals of the stress-exposed group in data set 2, perceived stress level was assessed using the visual analog scale (VAS) ranging from 0 (not at all stressed) to 100 (extremely stressed) with respect to their most traumatic experience throughout their career as a firefighter. Subclinical PTS symptom severity was evaluated using the CAPS^22,65^. A higher score on the VAS indicates that the individual perceived the relevant traumatic event to be more stressful, while a higher score on the CAPS represents a greater severity in PTS symptoms.

### Statistical analysis

For data set 1, GLMs were performed to examine differences in standardized mean connectivity strength of each inter-network connection cluster between the low-grade inflammatory and noninflammatory groups after adjusting for age and sex. For data set 2, differences in standardized mean connectivity strength of each inter-network cluster were examined between the low-grade inflammatory and noninflammatory groups, as well as between the stress-exposed and -unexposed groups, respectively, using GLMs. Age and sex were included as relevant covariates into the models. The reproducibility of the results was assessed by repeated analyses, including additional covariates such as FD and BMI in the statistical models (Supplementary Notes 1 and 2).

As post hoc analyses for data set 2, standardized mean connectivity strength of each inter-network connection cluster was compared between the noninflammatory group without stress exposure and each of the three groups, including the low-grade inflammation/stress-unexposed, no inflammation/stress-exposed, and low-grade inflammation/stress-exposed groups, respectively, after adjusting for age and sex. Permutation-adjusted *P* values for each model were calculated^66^. A total of 10,000 permutations were performed to obtain an empirical null distribution of the effects under the null hypothesis.

In addition, partial correlation analysis was conducted on the stress-exposed group of data set 2 to determine the relationship between the strength of functional connectivity for each network cluster and PTS symptom severity. Age and sex were included as covariates.

### Reporting summary

Further information on research design is available in the Nature Research Reporting Summary linked to this article.

## Supplementary information

Supplementary Information

Reporting Summary

## Data Availability

All relevant data underlying the major findings are available within the paper and Supplementary Information file. Source data underlying Table 1, Figs. 3, 4, and 6, Supplementary Tables 1, 3 through 7, and Supplementary Figs. 7 and 8 are provided as a Source Data file. The individual-level data that support the findings of this study are available upon reasonable request from the corresponding author. Due to ethical and legal restrictions, individual-level data of this study cannot be made publicly available. Request to access raw data of individual-level data will be considered in relation to the relevant consents, rules, and regulations, and can be made via the Ewha Brain Institute Review Committee at e600134@ewha.ac.kr after print publication of this paper. Assurance of proper credentials for handling sensitive data will be required from the applicant(s) prior to data sharing. Approval by the Ewha Brain Institute Review Committee will be followed by transfer of data upon signed agreement between Ewha Brain Institute and applicant(s). Applicant(s) may be requested to provide reimbursement of data management or preparation costs, as the Ewha Brain Institute does not receive funding for processes involving data sharing, such as analyses required for the de-identification of data and preparation of protected access.

## Source data

Source Data

## Code availability

The FMRIB Software Library tools (FSL) used in this study is an open-source software available at http://www.fmrib.ox.ac.uk/fsl.
