# Supplementary material for: A double-hit of stress and low-grade inflammation on functional brain network mediates posttraumatic stress symptoms
Source: Nat Commun. 2020 Apr 20;11:1898. doi: 10.1038/s41467-020-15655-5 (PMC7171097; doi:10.1038/s41467-020-15655-5)
Supplement: Supplementary file 1 — Supplementary Information [file 41467_2020_15655_MOESM1_ESM.pdf]

A double-hit of stress and low-grade inflammation on functional brain network mediates posttraumatic stress symptoms

Jungyoon Kim et al.

Supplementary Note 1

Repeated analyses with the inclusion of potential confounding factors of head motion as measured by framewise displacement (FD) and body mass index (BMI) as an additional covariate, respectively.

Data set 1

<u>Analyses including head motion as an additional covariate:</u> The standardized mean connectivity strengths between the salience network (SAN)-central executive network (CEN)-anterior default mode network (DMN_a) showed significant differences between the low-grade inflammatory and non-inflammatory groups, even after the additional adjustment for FD (generalized linear model [GLM], z = -2.76, permutation-adjusted P = 0.006). Similarly, there was a significant between-group difference in connectivity strength of the inter-network cluster 1 after adjusting for FD (GLM, z = -2.40, permutation-adjusted P = 0.02). The directionality of between-group differences in other inter-network connection clusters also did not change after the additional adjustment for FD (*a priori-defined inter-network clustering*: SAN-DMN_a, GLM, z = -1.24, permutation-adjusted P = 0.21; SMN-CEN-VIN, GLM, z = 1.45, permutation-adjusted P = 0.15; SMN-SAN-VIN, GLM, z = 0.01, permutation-adjusted P = 0.99; SMN-DMN-VIN, GLM, z = 1.86, permutation-adjusted P = 0.07)(*data-driven inter-network clustering*: inter-network connection cluster 2, GLM, z = -2.09, permutation-adjusted P = 0.04; inter-network connection cluster 3, GLM, z = 2.17, permutation-adjusted P = 0.03; inter-network connection cluster 4, GLM, z = 0.27, permutation-adjusted P = 0.78; inter-network connection cluster 5, GLM, z = 1.43, permutation-adjusted P = 0.16)

<u>Analyses including BMI as an additional covariate:</u> Standardized mean connectivity strengths of the inter-network connection clusters were compared between the low-grade inflammatory and non-inflammatory groups with BMI included as an additional covariate. The results remained similar to those from the original model (*a priori-defined inter-network clustering*: SAN-CEN-DMN_a, GLM, z = -2.68, permutation-adjusted P = 0.008; SAN-DMN_a, GLM, z = -1.87, permutation-adjusted P = 0.07; SMN-CEN-VIN, GLM, z = 1.46, permutation-adjusted P = 0.15; SMN-SAN-VIN, GLM, z = 0.22, permutation-adjusted P = 0.82; SMN-DMN-VIN, GLM, z = 2.46, permutation-adjusted P = 0.01)(*data-driven inter-network clustering*: inter-network connection cluster 1, GLM, z = -2.32, permutation-adjusted P = 0.02; inter-network connection cluster 2, GLM, z = -2.71, permutation-adjusted P = 0.009; inter-network connection cluster 3, GLM, z = 2.24, permutation-adjusted P = 0.03; inter-network connection cluster 4, GLM, z = 0.63, permutation-adjusted P = 0.52; inter-network connection cluster 5, GLM, z = 1.97, permutation-adjusted P = 0.049).

Data set 2

<u>Analyses including head motion as an additional covariate:</u> The standardized mean connectivity strengths of the inter-network connection clusters were compared between the low-grade inflammatory and non-inflammatory groups after the additional adjustment for head motion as measured in FD values. With the initial analyses without the adjustment for head motion as references, similar results were obtained in all inter-network connection clusters (*a priori-defined inter-network clustering*: SAN-CEN-DMN_a, GLM, z = -2.70, permutation-adjusted P = 0.008; SAN-DMN_a, GLM, z = -1.02, permutation-adjusted P = 0.31; SMN-CEN-VIN, GLM, z = 0.54, permutation-adjusted P = 0.60; SMN-SAN-VIN, GLM, z = -0.07, permutation-adjusted P = 0.94; SMN-DMN-VIN, GLM, z = 1.92, permutation-adjusted P = 0.06)(*data-driven inter-network clustering*: inter-network connection cluster 1, GLM, z = -2.67, permutation-adjusted P = 0.007; inter-network connection cluster 2, GLM, z = -2.34, permutation-adjusted P = 0.02; inter-network connection cluster 3, GLM, z = 0.60, permutation-adjusted P = 0.56; inter-network connection cluster 4, GLM, z = 0.09, permutation-adjusted P = 0.93; inter-network connection cluster 5, GLM, z = 1.56, permutation-adjusted P = 0.12).

The differences in standardized mean connectivity strengths of the inter-network connection clusters between the stress-exposed and stress-unexposed groups also remained unchanged after the additional adjustment for FD (*a priori-defined inter-network clustering*: SAN-CEN-DMN_a, GLM, z = -1.05, permutation-adjusted P = 0.31; SAN-DMN_a, GLM, z = -2.23, permutation-adjusted P = 0.03; SMN-CEN-VIN, GLM, z = 0.67, permutation-adjusted P = 0.51; SMN-SAN-VIN, GLM, z = -3.27, permutation-adjusted P = 0.002; SMN-DMN-VIN, GLM, z = 0.48, permutation-adjusted P = 0.63)(*data-driven inter-network clustering*: inter-network connection cluster 1, GLM, z = -1.02, permutation-adjusted P = 0.32; inter-network connection cluster 2, GLM, z = -3.23, permutation-adjusted P = 0.002; inter-network connection cluster 3, GLM, z = 1.04, permutation-adjusted P = 0.31; inter-network connection cluster 4, GLM, z = -2.77, permutation-adjusted P = 0.008; inter-network connection cluster 5, GLM, z = -0.78, permutation-adjusted P = 0.44).

<u>Analyses including BMI as an additional covariate:</u> We also compared the inter-network connectivity strengths between the low-grade inflammatory and non-inflammatory groups of data set 2 with BMI as an additional covariate. These repeated analyses yielded results similar to the initial model as well (*a priori-defined inter-network clustering*: SAN-CEN-DMN_a, GLM, z = -2.57, permutation-adjusted P = 0.01; SAN-DMN_a, GLM, z = -1.48, permutation-adjusted P = 0.14; SMN-CEN-VIN, GLM, z = 0.17, permutation-adjusted P = 0.87; SMN-SAN-VIN, GLM, z = 0.26, permutation-adjusted P = 0.79; SMN-DMN-VIN, GLM, z = 2.49, permutation-adjusted P = 0.01)(*data-driven inter-network clustering*: inter-network connection cluster 1, GLM, z = -2.50, permutation-adjusted P = 0.01; inter-network connection cluster 2, GLM, z = -2.80, permutation-adjusted P = 0.006; inter-network connection cluster 3, GLM, z = 0.28, permutation-adjusted P = 0.79; inter-network connection cluster 4, GLM, z = 0.56, permutation-adjusted P = 0.58; inter-network connection cluster 5, GLM, z = 2.08, permutation-adjusted P = 0.04).

Standardized mean connectivity strengths in the inter-network connection clusters were also compared between the stress-exposed and stress-unexposed groups

after adjusting for BMI and similar results were produced (*a priori-defined inter-network clustering*: SAN-CEN-DMN_a, GLM, z = -0.93, permutation-adjusted P = 0.37; SAN-DMN_a, GLM, z = -2.40, permutation-adjusted P = 0.02; SMN-CEN-VIN, GLM, z = 0.55, permutation-adjusted P = 0.59; SMN-SAN-VIN, GLM, z = -2.83, permutation-adjusted P = 0.007; SMN-DMN-VIN, GLM, z = 0.79, permutation-adjusted P = 0.43)(*data-driven inter-network clustering*: inter-network connection cluster 1, GLM, z = -0.90, permutation-adjusted P = 0.38; inter-network connection cluster 2, GLM, z = -3.37, permutation-adjusted P = 0.0005; inter-network connection cluster 3, GLM, z = 0.92, permutation-adjusted P = 0.38; inter-network connection cluster 4, GLM, z = -2.19, permutation-adjusted P = 0.03; inter-network connection cluster 5, GLM, z = -0.35, permutation-adjusted P = 0.72).

Supplementary Note 2

Exploratory analyses were conducted to ensure the robustness of the current findings across various network node dimensionalities. We first examined whether the patterns of the functional coupling of network nodes were comparable across the 3 distinct inter-network connection matrices according to its dimensionality (25 vs. 77 determined by automatic estimation vs. 200). The Fisher z-transformed connectivity values for each inter-network edge that connect each pair of network nodes across 3 distinct inter-network connection matrices are presented in Supplementary Figure 6. The pattern of functional coupling of the major representative networks were similar across the 3 dimensionalities, which is consistent with previous studies[1,2]. In addition, we also found that decomposition with a higher dimensionality may essentially yield the same system as that of a lower dimensionality, with the sole exception that a higher dimensional decomposition splits or fractions the major representative networks obtained from a lower dimensional decomposition. The averaged connectivity values of the inter-network edges among the major representative networks including the CEN, SAN, DMN, SMN, and VIN, showed a similar pattern of functional coupling across the 3 dimensionalities (Supplementary Table 7). Furthermore, significant correlations of the averaged connectivity values among the major representative networks were observed across the 3 dimensionalities (13 vs. 29 matrices; 29 vs. 78 matrices; 13 vs. 78 matrices, respectively)(Supplementary Table 7).

We also examined whether the results from the group differences in inter-network connectivity strength were similar across the 3 distinct inter-network connection matrices in each data set. For data set 1, the connectivity strength was compared between the low-grade inflammatory and non-inflammatory groups at each inter-network edge level (Supplementary Figure 7a). In addition, the between-group differences in the averaged connectivity strength among the CEN, SAN, DMN, SMN, and VIN were also examined using generalized linear models (Supplementary Figure 7b). A similar pattern was observed for the group differences in the averaged connectivity strength across the 3 connection matrices. Specifically, significant between-group differences were found in the averaged connectivity strength of the CEN-DMN_a (13 matrix, GLM, z = -3.27, P = 0.001; 29 matrix, GLM, z = -3.31, P = 0.001; 78 matrix, GLM, z = -2.49, P = 0.01) as well as SAN-DMN_a (13 matrix, GLM, z = -1.98, P = 0.047; 29 matrix, GLM, z = -3.05, P = 0.002; 78 matrix, GLM, z = -2.81, P = 0.005) in all 3 inter-network connection matrices. In addition, connection strength between the SMN-DMN-VIN demonstrated to be enhanced in the low-grade inflammatory group relative to the non-inflammatory group in all 3 connection matrices (13 matrix, GLM, z = 2.44, P = 0.02; 29 matrix, GLM, z = 2.31, P = 0.02; 78 matrix, GLM, z = 1.81, P = 0.07). There were no significant differences in connection strength of the SMN-CEN-VIN (13 matrix, GLM, z = 1.03, P = 0.31; 29 matrix, GLM, z = 1.34, P = 0.18; 78 matrix, GLM, z = 1.81, P = 0.07), SMN-SAN-VIN (13 matrix, GLM, z = 0.59, P = 0.56; 29 matrix, GLM, z = -1.55, P = 0.12; 78 matrix, GLM, z = -1.26, P = 0.21), and CEN-SAN (13 matrix, GLM, z = -1.08, P = 0.28; 29 matrix, GLM, z = -0.75, P = 0.45; 78 matrix, GLM, z = -1.31, P = 0.19) between the low-grade inflammatory and non-inflammatory groups in all 3 connection matrices.

For data set 2, the connectivity strengths among the major representative

networks were compared between the stress-exposed and stress-unexposed groups across the 3 inter-network connection matrices with various dimensionalities. As observed in data set 1, a similar pattern of group differences in inter-network connectivity strength was found at each inter-network edge level across the 3 connection matrices (Supplementary Figure 8a). Standardized differences in connectivity strength are depicted in Supplementary Figure 8b. Specifically, the averaged connectivity strength of SAN-DMN_a (13 matrix, GLM, z = -2.34, P = 0.02; 29 matrix, GLM, z = -2.89, P = 0.004; 78 matrix, GLM, z = -1.96, P = 0.05) and SMN-SAN-VIN (13 matrix, GLM, z = -2.92, P = 0.003; 29 matrix, GLM, z = -3.30, P = 0.001; 78 matrix, GLM, z = -1.69, P = 0.09) were reduced in the stress-exposed group as compared to the stress-unexposed group across the 3 connection matrices, while it is noteworthy that the results from the 78 X 78 matrix did not reach statistical significance. There were no significant between-group differences in averaged connectivity strength of CEN-SAN (13 matrix, GLM, z = -0.42, P = 0.67; 29 matrix, GLM, z = -0.31, P = 0.76; 78 matrix, GLM, z = 0.02, P = 0.99), CEN-DMN_a (13 matrix, GLM, z = -0.73, P = 0.46; 29 matrix, GLM, z = -0.55, P = 0.58; 78 matrix, GLM, z = -0.67, P = 0.50), SMN-CEN-VIN (13 matrix, GLM, z = 0.51, P = 0.61; 29 matrix, GLM, z = 1.19, P = 0.23; 78 matrix, GLM, z = 0.54, P = 0.59), and SMN-DMN-VIN (13 matrix, GLM, z = 0.99, P = 0.32; 29 matrix, GLM, z = 1.39, P = 0.16; 78 matrix, GLM, z = -0.17, P = 0.87) in all 3 connection matrices.

Supplementary Table 1 Demographic and clinical characteristics of subgroups in each data set

Characteristics	Data set 1		Data set 2			
	No inflammation (n = 350)	Low-grade inflammation (n = 350)	Stress-unexposed/ no inflammation (n = 24)	Stress-unexposed/ low-grade inflammation (n = 22)	Stress exposed/ no inflammation (n = 25)	Stress-exposed/ low-grade inflammation (n = 27)
Age, mean (SD), y	33.3 (11.8)	32.0 (11.4)	32.7 (4.0)	33.3 (3.7)	33.4 (4.3)	33.8 (3.8)
Male, n (%)	203 (58.0)	207 (59.1)	21 (87.5)	18 (81.8)	21 (84.0)	24 (88.9)
Body mass index, mean (SD), kg/m^2	22.8 (2.5)	22.9 (2.7)	23.8 (2.2)	23.7 (2.4)	24.2 (2.0)	23.6 (2.5)
Inflammatory activity						
IL-6, mean (SD), pg/mL	2.81 (1.41)	6.58 (3.12)	2.40 (1.16)	6.61 (3.74)	2.61 (1.54)	6.14 (2.75)
IL-1β, mean (SD), pg/mL	1.92 (0.84)	3.89 (1.57)	1.59 (0.54)	2.96 (1.09)	1.45 (0.55)	2.86 (0.65)
TNF-α, mean (SD), pg/mL	9.62 (1.70)	12.58 (2.25)	9.77 (2.08)	12.02 (1.97)	9.71 (1.96)	12.38 (2.25)
Inflammatory composite[a], mean (SD), z score	-0.622 (0.349)	0.622 (0.645)	-0.592 (0.357)	0.633 (0.629)	-0.634 (0.366)	0.597 (0.411)

[a] Inflammatory composite values were calculated by averaging the sex-specific standardized z scores of the 3 pro-inflammatory cytokine levels. Source data are provided as a Source Data file.

Abbreviations: y, year; SD, standard deviation; IL-6, interleukin-6; IL-1β, interleukin-1β; TNF-α, tumor necrosis factor-α.

Supplementary Table 2 Rotated factor loadings for inter-network connection components

Inter-network connection cluster	Inter-network connection component	Functional connectome of each inter-network connection component	Inter-network functional coupling between network nodes	Factor loading
Inter-network connection cluster 1	Component 10	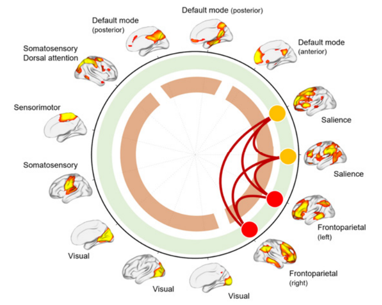	salience1-frontoparietal (right)<br>salience1-frontoparietal (left)<br>salience2-frontoparietal (right)<br>salience2-frontoparietal (left)<br>salience1-salience2<br>frontoparietal (right)-frontoparietal (left)	0.433<br>0.599<br>0.526<br>0.522<br>0.529<br>0.406
	Component 11	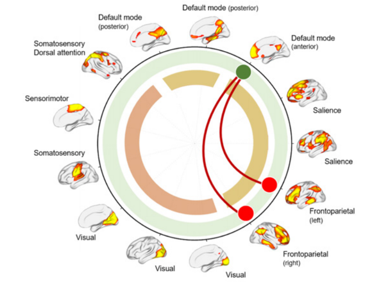	default mode (anterior)-frontoparietal (right)<br>default mode (anterior)-frontoparietal (left)	0.743<br>0.689
Inter-network connection cluster 2	Component 2	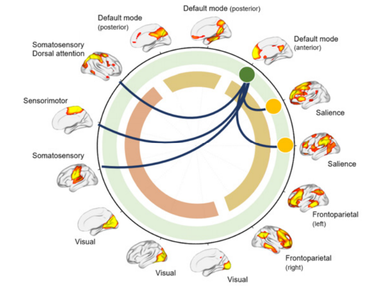	default mode (anterior)-salience1<br>default mode (anterior)-salience2<br>default mode (anterior)-somatosensory1<br>default mode (anterior)-sensorimotor<br>default mode (anterior)-somatosensory2	0.721<br>0.617<br>0.677<br>0.766<br>0.807
Inter-network connection cluster 3	Component 5	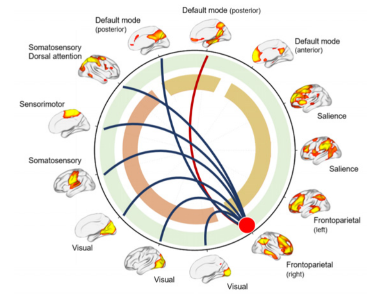	frontoparietal (right)-visual1<br>frontoparietal (right)-visual2<br>frontoparietal (right)-visual3<br>frontoparietal (right)-somatosensory1<br>frontoparietal (right)-sensorimotor<br>frontoparietal (right)-somatosensory2<br>frontoparietal (right)-default mode (posterior1)<br>frontoparietal (right)-default mode (posterior2)	0.838<br>0.697<br>0.666<br>0.613<br>0.712<br>0.610<br>0.685<br>0.628
	Component 6	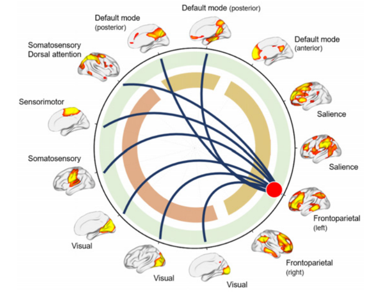	frontoparietal (left)-visual1<br>frontoparietal (left)-visual2<br>frontoparietal (left)-visual3<br>frontoparietal (left)-somatosensory1<br>frontoparietal (left)-sensorimotor<br>frontoparietal (left)-somatosensory2<br>frontoparietal (left)-default mode (posterior1)<br>frontoparietal (left)-default mode (posterior2)	0.850<br>0.721<br>0.597<br>0.604<br>0.715<br>0.587<br>0.732<br>0.644

(continued on next page)

Inter-network connection cluster	Inter-network connection component	Functional connectome of each inter-network connection component	Inter-network functional coupling between network nodes	Factor loading
Inter-network connection cluster 4	Component 4	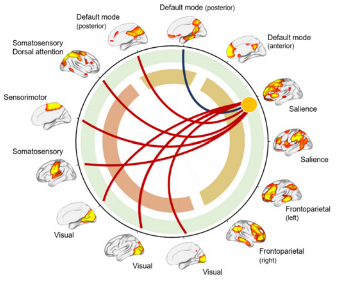	salience2-visual1<br>salience2-visual2<br>salience2-visual3<br>salience2-somatosensory1<br>salience2-sensorimotor<br>salience2-somatosensory2<br>salience2-default mode (posterior1)<br>salience2-default mode (posterior2)	0.857<br>0.693<br>0.708<br>0.690<br>0.752<br>0.716<br>0.638<br>0.607
	Component 8	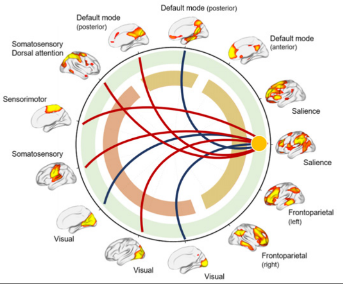	salience1-visual1<br>salience1-visual2<br>salience1-visual3<br>salience1-somatosensory1<br>salience1-sensorimotor<br>salience1-somatosensory2<br>salience1-default mode (posterior1)<br>salience1-default mode (posterior2)	0.852<br>0.627<br>0.669<br>0.499<br>0.697<br>0.614<br>0.603<br>0.556
Inter-network connection cluster 5	Component 1	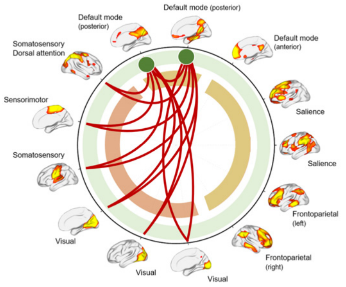	default mode (posterior1)-visual1<br>default mode (posterior1)-visual2<br>default mode (posterior1)-visual3<br>default mode (posterior1)-somatosensory1<br>default mode (posterior1)-sensorimotor<br>default mode (posterior1)-somatosensory2<br>default mode (posterior1)-default mode (posterior2)<br>default mode (posterior2)-visual1<br>default mode (posterior2)-visual2<br>default mode (posterior2)-visual3<br>default mode (posterior2)-somatosensory1<br>default mode (posterior2)-sensorimotor<br>default mode (posterior2)-somatosensory2	0.784<br>0.673<br>0.600<br>0.570<br>0.601<br>0.525<br>0.586<br>0.728<br>0.684<br>0.566<br>0.564<br>0.545<br>0.530
	Component 9	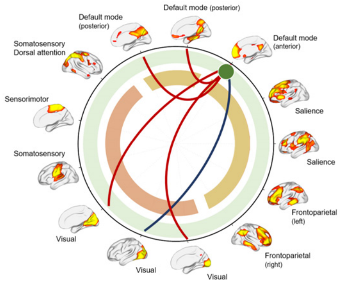	default mode (anterior)-visual1<br>default mode (anterior)-visual2<br>default mode (anterior)-visual3<br>default mode (anterior)-default mode (posterior1)<br>default mode (anterior)-default mode (posterior2)	0.811<br>0.575<br>0.732<br>0.772<br>0.700

(continued on next page)

Inter-network connection cluster	Inter-network connection component	Functional connectome of each inter-network connection component	Inter-network functional coupling between network nodes	Factor loading
NA	Component 3	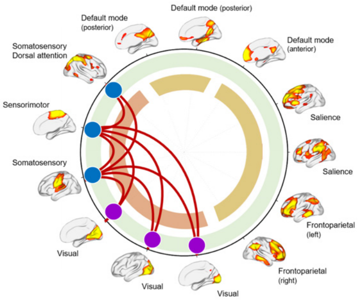	sensorimotor-somatosensory1<br>sensorimotor-somatosensory2<br>sensorimotor-visual1<br>sensorimotor-visual2<br>sensorimotor-visual3<br>somatosensory2-somatosensory1<br>somatosensory2-visual1<br>somatosensory2-visual2<br>somatosensory2-visual3	0.402<br>0.432<br>0.690<br>0.646<br>0.741<br>0.435<br>0.766<br>0.602<br>0.744
	Component 7	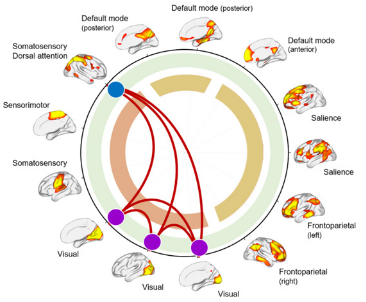	somatosensory1-visual1<br>somatosensory1-visual2<br>somatosensory1-visual3<br>visual1-visual2<br>visual1-visual3<br>visual2-visual3	0.539<br>0.396<br>0.624<br>0.607<br>0.650<br>0.716

Abbreviations: NA, not available.

Supplementary Table 3. Group differences in standardized mean connectivity strength of inter-network connection clusters in data set 1

	Standardized mean connectivity strength (95% CI)		*z*	*P*
	No inflammation (n = 350)	Low-grade inflammation (n = 350)		
A priori-defined inter-network connection clusters				
SAN-CEN-DMN_a	0.00 (-0.110 to 0.110)	-0.207 (-0.305 to -0.108)	-2.66	0.008
SAN-DMN_a	0.00 (-0.109 to 0.109)	-0.150 (-0.251 to -0.049)	-1.89	0.06
SMN-CEN-VIN	0.00 (-0.113 to 0.113)	0.078 (-0.019 to 0.175)	1.44	0.15
SMN-SAN-VIN	0.00 (-0.108 to 0.108)	0.045 (-0.058 to 0.147)	0.27	0.79
SMN-DMN-VIN	0.00 (-0.102 to 0.102)	0.184 (0.077 to 0.291)	2.48	0.01
Data-driven inter-network connection clusters				
Inter-network cluster 1	0.00 (-0.112 to 0.112)	-0.179 (-0.275 to -0.083)	-2.31	0.02
Inter-network cluster 2	0.00 (-0.106 to 0.106)	-0.214 (-0.317 to -0.111)	-2.72	0.008
Inter-network cluster 3	0.00 (-0.111 to 0.111)	0.137 (0.039 to 0.236)	2.22	0.03
Inter-network cluster 4	0.00 (-0.104 to 0.104)	0.077 (-0.029 to 0.184)	0.69	0.49
Inter-network cluster 5	0.00 (-0.101 to 0.101)	0.144 (0.035 to 0.253)	1.98	0.049

Generalized linear models were used to examine group differences in standardized mean connectivity strength for each inter-network connection cluster. For all analyses, age and sex composition were included into the statistical models as covariates. *P* values (for two-sided testing) were computed using 10,000 permutations. Source data are provided as a Source Data file.

Abbreviations: CI, confidence interval; SAN, salience network; DMN, default mode network; DMN_a, default mode network, anterior; CEN, central executive network; SMN, sensorimotor network; VIN, visual network.

Supplementary Table 4. Group differences in standardized mean connectivity strength of inter-network connection clusters in data set 2

Inter-network connection cluster	Standardized mean connectivity strength (95% CI)		*z*	*P*
	No inflammation (n = 49)	Low-grade inflammation (n = 49)		
A priori-defined inter-network connection clusters				
SAN-CEN-DMN$_a$	0.063 (-0.185 to 0.311)	-0.410 (-0.719 to -0.101)	-2.53	0.01
SAN-DMN$_a$	-0.172 (-0.454 to 0.109)	-0.433 (-0.724 to -0.142)	-1.35	0.18
SMN-CEN-VIN	0.017 (-0.252 to 0.286)	0.082 (-0.225 to 0.389)	0.33	0.75
SMN-SAN-VIN	-0.323 (-0.616 to -0.031)	-0.293 (-0.577 to -0.008)	0.26	0.79
SMN-DMN-VIN	0.093 (-0.154 to 0.339)	0.560 (0.249 to 0.871)	2.27	0.02
Data-driven inter-network connection clusters				
Inter-network cluster 1	0.063 (-0.189 to 0.314)	-0.390 (-0.698 to -0.082)	-2.48	0.01
Inter-network cluster 2	-0.310 (-0.582 to -0.037)	-0.830 (-1.115 to -0.545)	-2.61	0.009
Inter-network cluster 3	0.066 (-0.196 to 0.328)	0.151 (-0.162 to 0.463)	0.41	0.70
Inter-network cluster 4	-0.294 (-0.582 to -0.005)	-0.204 (-0.493 to 0.085)	0.53	0.60
Inter-network cluster 5	-0.045 (-0.285 to 0.195)	0.358 (0.038 to 0.678)	1.93	0.05

Inter-network connection cluster	Standardized mean connectivity strength (95% CI)		*z*	*P*
	Stress-unexposed (n = 46)	Stress-exposed (n = 52)		
A priori-defined inter-network connection clusters				
SAN-CEN-DMN$_a$	-0.087 (-0.389 to 0.214)	-0.250 (-0.526 to 0.026)	-0.94	0.36
SAN-DMN$_a$	-0.057 (-0.296 to 0.183)	-0.520 (-0.828 to -0.212)	-2.41	0.02
SMN-CEN-VIN	-0.005 (-0.359 to 0.349)	0.098 (-0.125 to 0.322)	0.52	0.62
SMN-SAN-VIN	-0.006 (-0.263 to 0.252)	-0.575 (-0.865 to -0.285)	-2.84	0.006
SMN-DMN-VIN	0.220 (-0.072 to 0.513)	0.420 (0.138 to 0.702)	0.82	0.41
Data-driven inter-network connection clusters				
Inter-network cluster 1	-0.086 (-0.389 to 0.216)	-0.232 (-0.507 to 0.043)	-0.91	0.38
Inter-network cluster 2	-0.221 (-0.479 to 0.038)	-0.878 (-1.160 to -0.596)	-3.38	0.0005
Inter-network cluster 3	0.010 (-0.340 to 0.360)	0.195 (-0.031 to 0.421)	0.89	0.39
Inter-network cluster 4	-0.011 (-0.279 to 0.257)	-0.459 (-0.749 to -0.169)	-2.20	0.03
Inter-network cluster 5	0.171 (-0.112 to 0.455)	0.144 (-0.148 to 0.435)	-0.33	0.74

Generalized linear models were used to examine group differences in standardized mean connectivity strength for each inter-network connection cluster. For all analyses, age and sex composition were included into the statistical models as covariates. *P* values (for two-sided testing) were computed using 10,000 permutations. Source data are provided as a Source Data file.

Abbreviations: CI, confidence interval; SAN, salience network; DMN, default mode network; DMN$_a$, default mode network, anterior; CEN, central executive network; SMN, sensorimotor network; VIN, visual network.

Supplementary Table 5. Associations between standardized mean connectivity strength of inter-network connection clusters and PTS symptom severity

	Associations with PTS symptom severity	
	Partial correlation coefficients	*P*
A priori-defined inter-network connection clusters		
SAN-CEN-DMN_a	-0.33	0.02
SAN-DMN_a	-0.32	0.02
SMN-CEN-VIN	0.09	0.51
SMN-SAN-VIN	0.04	0.77
SMN-DMN-VIN	0.34	0.01
Data-driven inter-network connection clusters		
Inter-network cluster 1	-0.34	0.02
Inter-network cluster 2	-0.37	0.009
Inter-network cluster 3	0.14	0.33
Inter-network cluster 4	0.09	0.52
Inter-network cluster 5	0.29	0.04

Partial correlation analyses were performed to examine the associations between connectivity strengths of the inter-network connection clusters and PTS symptom severity. For all analyses, age and sex composition were included as covariates. Source data are provided as a Source Data file.

Abbreviations: PTS, posttraumatic stress; SAN, salience network; DMN, default mode network; DMN_a, default mode network, anterior; CEN, central executive network; SMN, sensorimotor network; VIN, visual network.

Supplementary Table 6. Associations between inter-network functional connectivity strength and each of CAPS subscale score

	Associations with reexperiencing symptom severity		Associations with avoidance symptom severity		Associations with hyperarousal symptom severity	
	Partial correlation coefficients	*P*	Partial correlation coefficients	*P*	Partial correlation coefficients	*P*
A priori-defined inter-network connection clusters						
SAN-CEN-DMN_a	-0.33	0.02	-0.08	0.60	-0.37	0.008
SAN-DMN_a	-0.25	0.08	-0.12	0.43	-0.35	0.01
SMN-CEN-VIN	-0.03	0.86	0.06	0.67	0.14	0.35
SMN-SAN-VIN	-0.005	0.97	-0.04	0.80	0.11	0.43
SMN-DMN-VIN	0.05	0.72	0.16	0.27	0.47	0.0006
Data-driven inter-network connection clusters						
Inter-network cluster 1	-0.30	0.03	-0.05	0.75	-0.42	0.002
Inter-network cluster 2	-0.20	0.16	-0.18	0.21	-0.41	0.003
Inter-network cluster 3	0.0007	1.00	0.13	0.37	0.15	0.30
Inter-network cluster 4	0.02	0.89	-0.002	0.99	0.16	0.26
Inter-network cluster 5	-0.003	0.98	0.11	0.46	0.44	0.001

Partial correlation analyses were performed to examine the associations between inter-network connectivity strengths of the inter-network connection clusters and CAPS subscale scores. For all analyses, age and sex composition were included as covariates. Source data are provided as a Source Data file.

Abbreviations: CAPS, the Clinician-Administered PTSD Scale for DSM-4; SAN, salience network; DMN, default mode network; DMN_a, default mode network, anterior; CEN, central executive network; SMN, sensorimotor network; VIN, visual network.

Supplementary Table 7. Averaged connectivity values among the major representative networks including the CEN, SAN, DMN, SMN, and VIN in each matrix based on group ICAs with various dimensionalities of 25, 77 (automatic estimated), and 200.

	13 by 13 matrix [Dimensionality of 25]	29 by 29 matrix [Dimensionality of 77[1]]	78 by 78 matrix [Dimensionality of 200]	Correlations between connectivity values[3]					
				13 vs. 29 matrices		13 vs. 78 matrices		29 vs. 78 matrices	
	Averaged connectivity values[2] (95% CI)	Averaged connectivity values[2] (95% CI)	Averaged connectivity values[2] (95% CI)	*r*	*P*	*r*	*P*	*r*	*P*
CEN-SAN	1.84 (1.75 to 1.94)	-0.07 (-0.14 to 0.01)	0.95 (0.92 to 0.99)	0.48	<0.0001	0.53	<0.0001	0.60	<0.0001
CEN-DMN_a	1.11 (0.99 to 1.24)	0.81 (0.75 to 0.87)	1.34 (1.31 to 1.36)	0.64	<0.0001	0.08	0.02	0.20	<0.0001
SAN-DMN_a	-2.17 (-2.34 to -2.01)	-0.90 (-1.03 to -0.78)	-0.55 (-0.62 to -0.49)	0.73	<0.0001	0.73	<0.0001	0.76	<0.0001
SMN-CEN-VIN	-1.25 (-1.35 to -1.15)	-0.42 (-0.48 to -0.37)	-0.43 (-0.46 to -0.39)	0.76	<0.0001	0.77	<0.0001	0.66	<0.0001
SMN-SAN-VIN	1.50 (1.39 to 1.60)	1.00 (0.93 to 1.08)	0.45 (0.40 to 0.49)	0.43	<0.0001	0.64	<0.0001	0.64	<0.0001
SMN-DMN-VIN	1.86 (1.74 to 1.97)	1.78 (1.68 to 1.87)	0.89 (0.85 to 0.93)	0.95	<0.0001	0.82	<0.0001	0.80	<0.0001

[1] Automatic estimation by the Bayesian dimensionality estimation technique yielded 77 components as an optimal dimensionality.
[2] Averaged Fisher z-transformed connectivity values of the inter-network edges that connect the major representative networks including the CEN, SAN, DMN, SMN, and VIN were calculated across a total of 798 individuals, which is the sum of both data sets 1 and 2. Fisher z-transformed connectivity values at every edge level are presented in Supplementary Figure 6.
[3] Pearson correlation analyses of the averaged connectivity values were performed among 3 distinct connection matrices (13 vs. 29; 13 vs. 78, and 29 vs. 78), respectively. Values of *r* in the table indicate Pearson correlation coefficients.

Source data are provided as a Source Data file.

Abbreviations: ICA, independent component analysis; CEN, central executive network; SAN, salience network; DMN, default mode network; DMN_a, default mode network, anterior; SMN, sensorimotor network; VIN, visual network; SMN, sensorimotor network; VIN, visual network; CI, confidence interval.

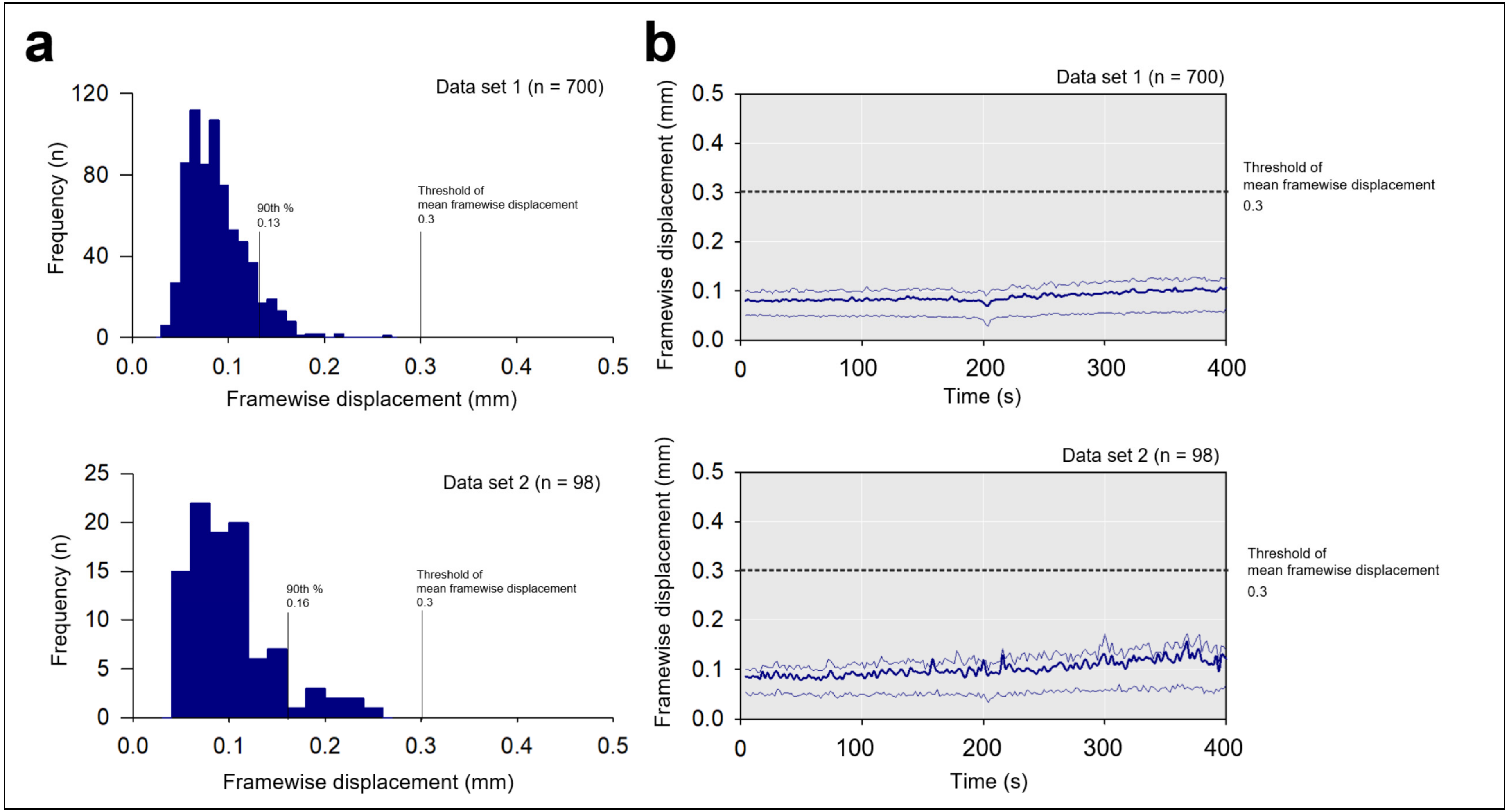

Supplementary Figure 1 Distribution of the mean framewise displacement (FD) **(a)** and mean FD trace **(b)** for data sets 1 and 2. In both data sets 1 and 2, the mean FD values of all images fell within the threshold of 0.3 mm and the head position was stable across the vast majority (90%) of the images, where the values were within 0.13 mm (data set 1) and 0.16 mm (data set 2), respectively. The thick blue lines in panel b indicate mean FD values while the thin blue lines represent the interquartile range. The dotted lines in panel b represent the mean FD threshold of 0.3 mm.

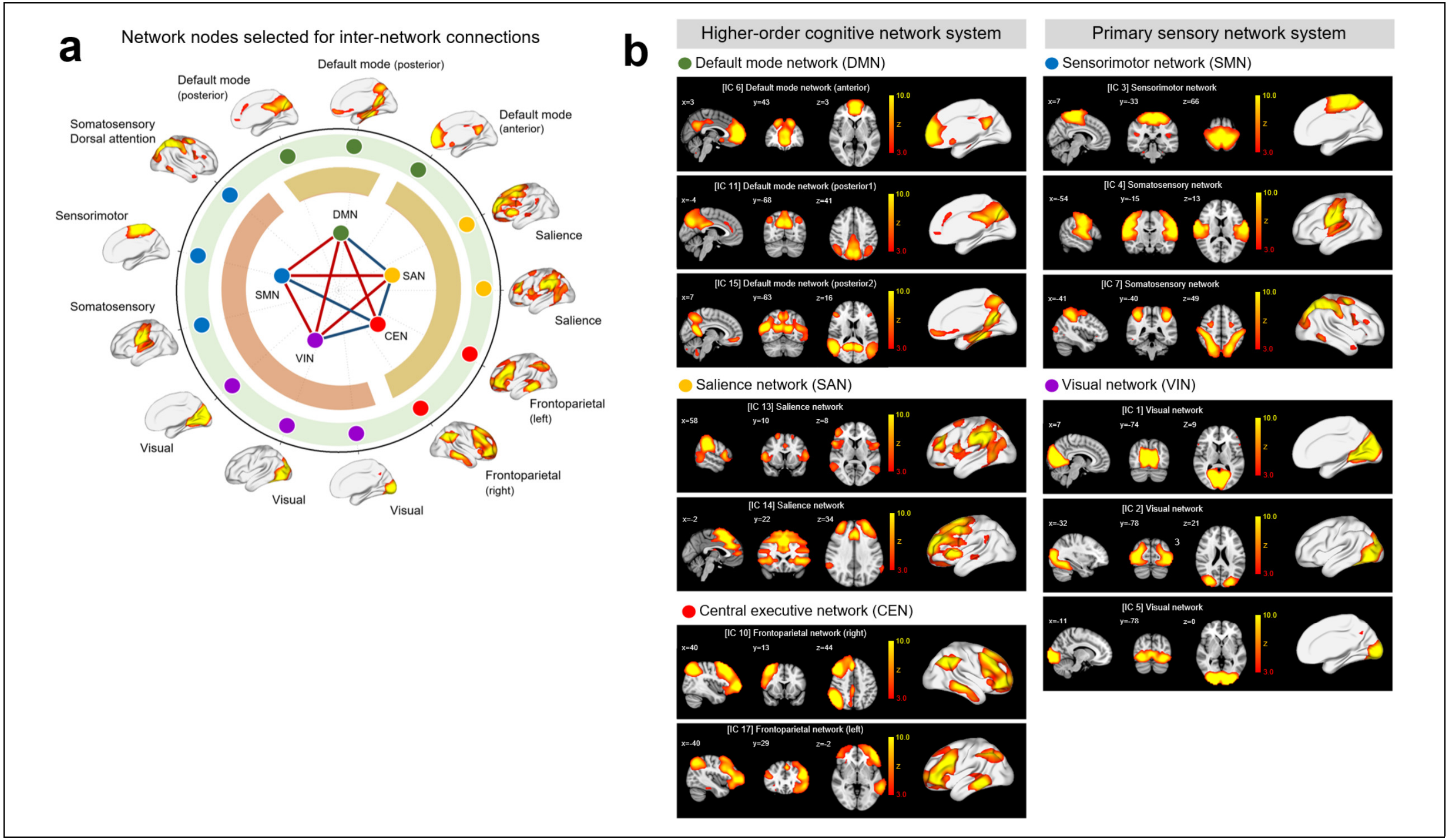

Supplementary Figure 2 A visual display of the network nodes and their spatial maps

a Group independent component analysis with 25 dimensionalities was performed on the processed data from data sets 1 and 2 (n = 798), from which 13 resting-state networks were selected as candidate network nodes for the generation of the inter-network functional connection matrix. The 13 candidate network nodes were further categorized into the CEN (red circles), SAN (yellow circles), DMN (green circles), SMN (blue circles), and VIN (purple circles).

b Spatial maps of z score images for the 13 network nodes identified from group ICA with a predetermined dimensionality of 25. Among the 25 independent components (ICs) based on the group ICA of 798 individuals, 13 resting-state network components were selected as group-averaged network nodes for the construction of the inter-network connection matrix. Spatial maps were converted to z score images and were thresholded at z = 3.0 (*P* = 0.001). The number of IC indicates the amount of variance explained by the corresponding IC in a decreasing order. These 13 network nodes were further grouped into 5 categories which are the DMN (green circles), SAN (yellow circle), CEN (red circles), SMN (blue circles), and VIN (purple circles). In the current study, we defined the DMN, SAN, and CEN as the higher-order cognitive network system while the VIN and SMN were defined as the primary sensory network system.

Abbreviations: ICA, independent component analysis; SAN, salience network; DMN, default mode network; CEN, central executive network; VIN, visual network; SMN, sensorimotor network.

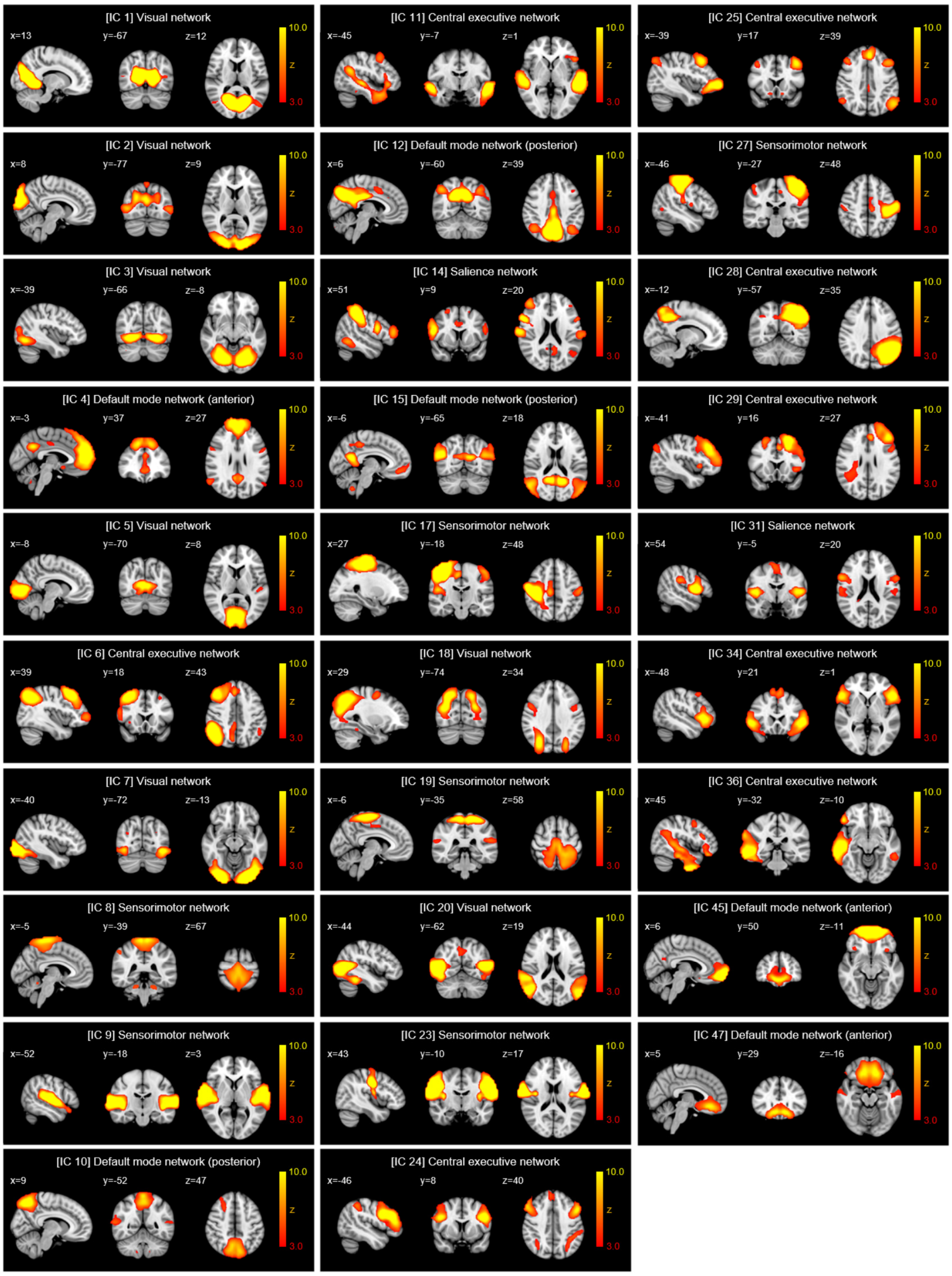

Supplementary Figure 3 Spatial maps of z score images for 29 network nodes from group ICA with an automatic determined dimensionality. Among the automatically determined 77 independent components (ICs) based on the group ICA of 798 individuals, brain network ICs that did not fall under the interests of the current study aims such as the cerebellum, subcortex, and temporal networks as well as noise ICs were removed. Finally, 29 resting-state network components were selected as group-averaged network nodes for the construction of the inter-network connection matrix. Spatial maps were converted to z score images and were thresholded at z = 3.0 (*P* = 0.001). The number of IC indicates the amount of variance explained by the corresponding IC in a decreasing order.

Abbreviations: ICA, independent component analysis; SAN, salience network; DMN, default mode network; CEN, central executive network; VIN, visual network; SMN, sensorimotor network.

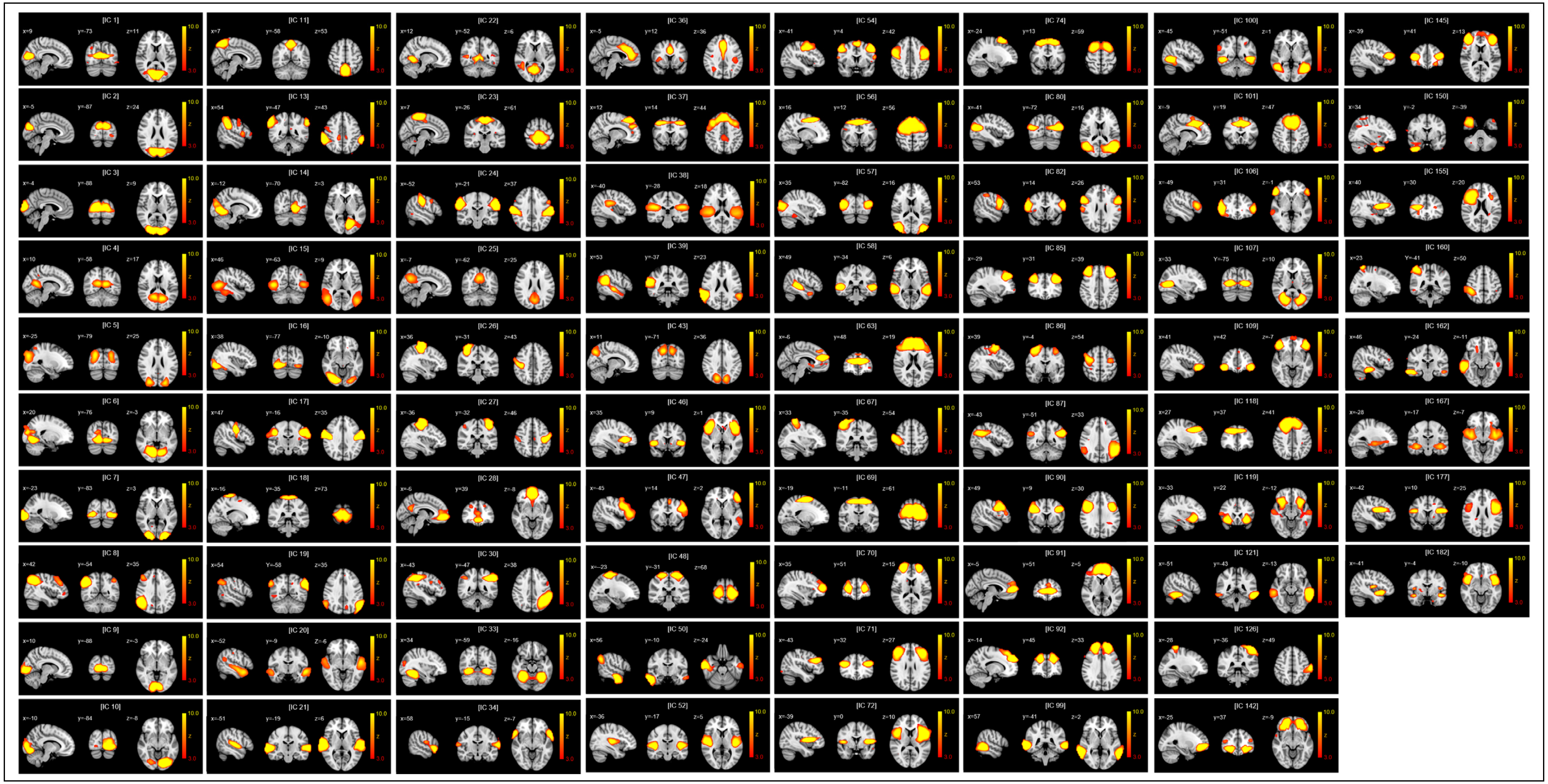

Supplementary Figure 4 Spatial maps of z score images for 78 network nodes from group ICA with a predetermined dimensionality of 200. Among the 200 independent components (ICs) based on the group ICA of 798 individuals, brain network ICs that did not fall under the interests of the current study aims such as the cerebellum, subcortex, and temporal networks as well as noise ICs were removed. Finally, 78 resting-state network components were selected as group-averaged network nodes for the construction of the inter-network connection matrix. Spatial maps were converted to z score images and were thresholded at $z = 3.0$ ($P = 0.001$). The number of IC indicates the amount of variance explained by the corresponding IC in a decreasing order.

Abbreviations: ICA, independent component analysis; SAN, salience network; DMN, default mode network; CEN, central executive network; VIN, visual network; SMN, sensorimotor network.

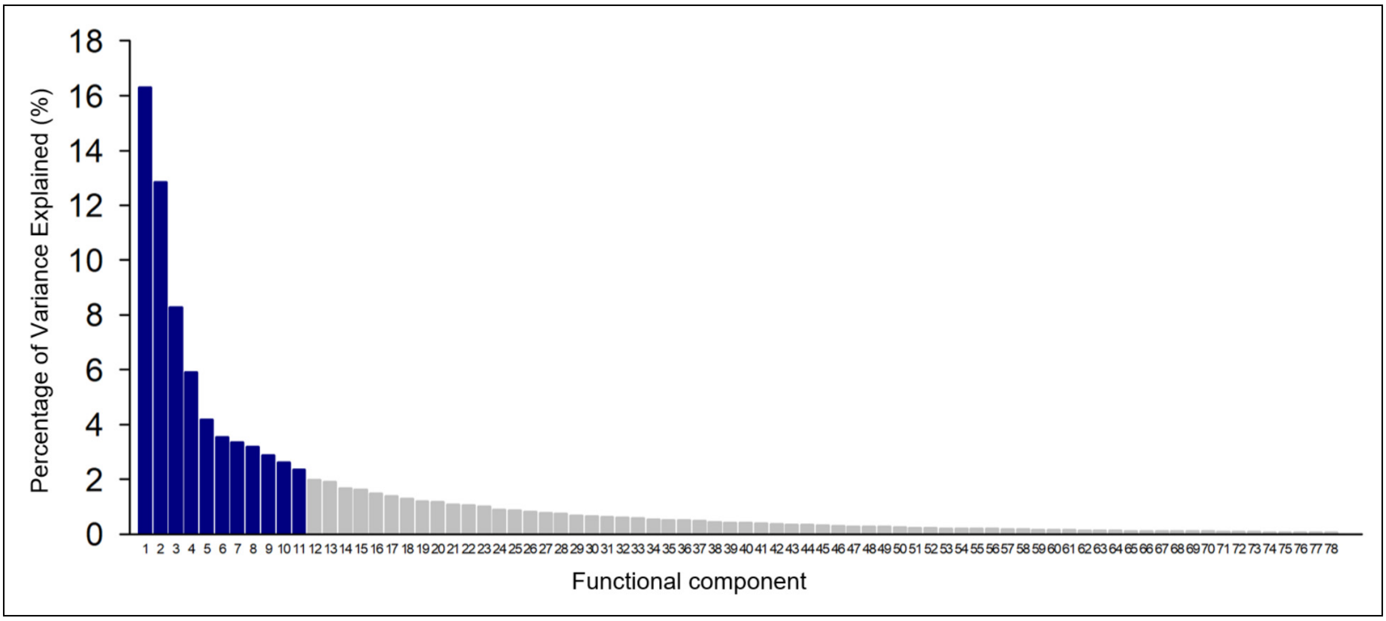

Supplementary Figure 5 Bar graph for percentage of variances that were explained by each functional component for the subset of network edges. The first 11 components selected in this study can explain 65.5% of total variance.

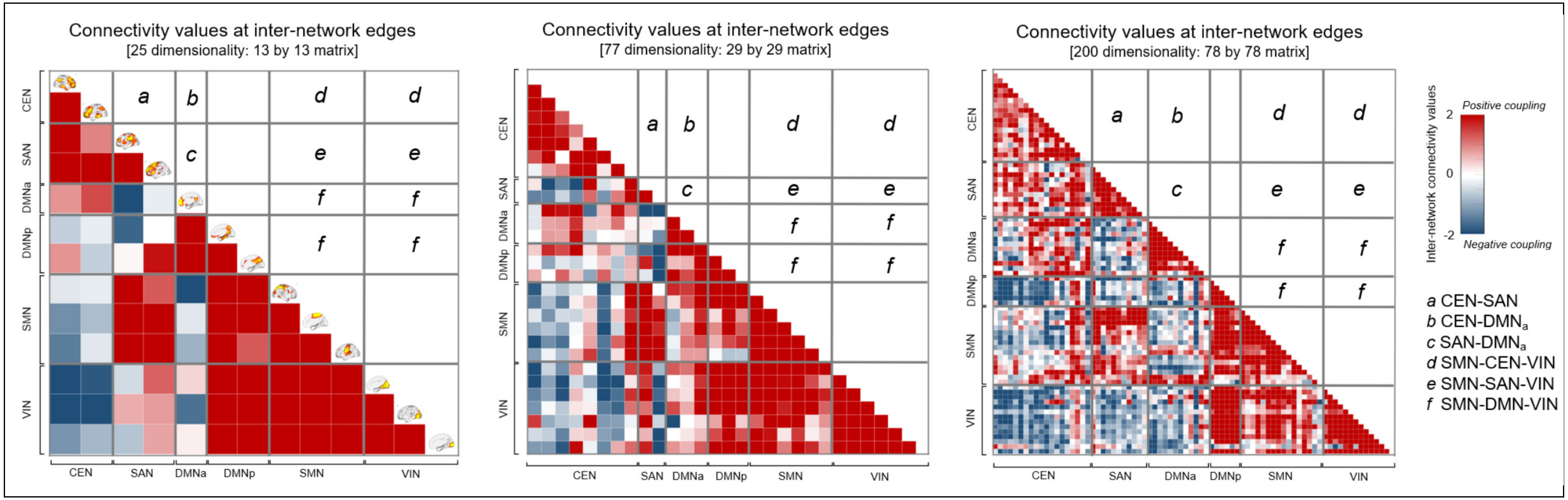

Supplementary Figure 6 Fisher z-transformed connectivity values at an inter-network edge-level were averaged across 798 individuals in the inter-network connection matrices with dimensionalities of 25 (left), 77 (automatic estimated, center), and 200 (right). Red and blue colors indicate positive and negative couplings of network node pairs, respectively. A darker color represents stronger inter-network connections.

Abbreviations: CEN, central executive network; SAN, salience network; DMN, default mode network; DMN_a, default mode network, anterior; DMN_p, default mode network, posterior; SMN, sensorimotor network; VIN, visual network..

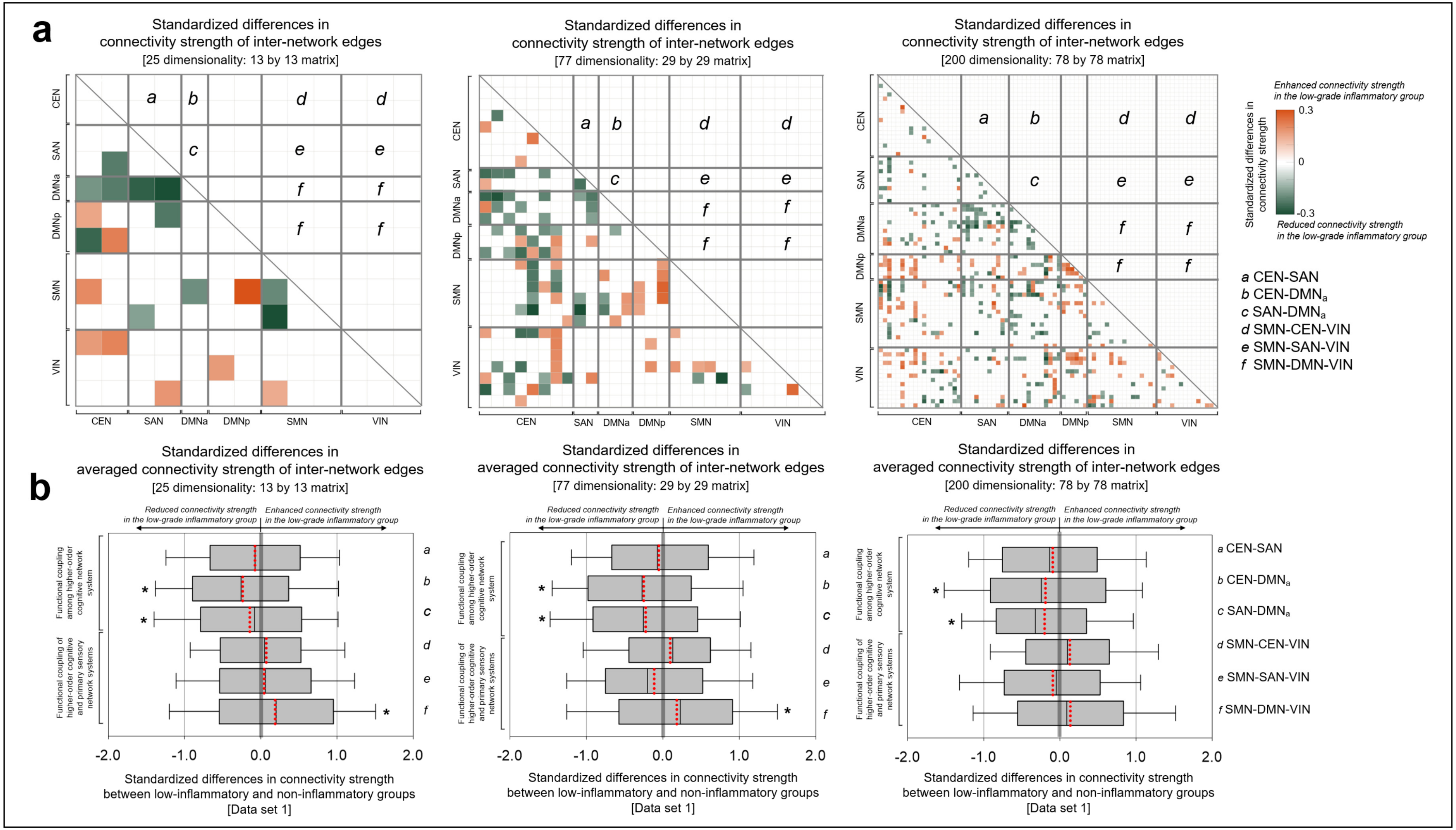

Supplementary Figure 7 Values of matrices with dimensionalities of 25 (left), 77 (automatic estimation, center), and 200 (right) indicating the standardized differences in connectivity strength at an inter-network edge-level between the low-grade inflammatory (n =350) and non-inflammatory (n =350) groups of data set 1 at a statistical significance of *P* < 0.05 (**a**) and box and whisker plots representing the distribution of standardized differences in averaged connectivity strength among inter-network edges that connect the network nodes (*a* CEN-SAN; *b* CEN-DMN$_a$; *c* SAN-DMN$_a$; *d* SMN-CEN-VIN; *e* SMN-SAN-VIN; *f* SMN-DMN-VIN) between the low-grade inflammatory (n =350) and non-inflammatory (n =350) groups of data set 1. (**b**).

Standardized between-group differences in connectivity strength were calculated by using the means and standard deviations for inter-network

connectivity strength of the non-inflammatory group. Positive and negative values indicate enhanced and reduced connectivity strength, respectively, in the low-grade inflammatory group as compared to the non-inflammatory group. Orange and green colors in the panel a indicate enhanced and reduced connectivity strength in the low-grade inflammatory group relative to the non-inflammatory group, respectively. The composition of inter-network edges, which constitute each block of the matrices (*a* CEN-SAN; *b* CEN-DMN_a; *c* SAN-DMN_a; *d* SMN-CEN-VIN ; *e* SMN-SAN-VIN; *f* SMN-DMN-VIN) was similar but not identical to the composition of inter-network connection clusters derived from the factor analyses in the main findings. Asterisks (*) in panel b indicate statistically significant between-group differences in averaged connectivity strength among inter-network edges at $P < 0.05$ (for the CEN-$DMN_{a,}$, 13 matrix, $z = -3.27$, $P = 0.001$; 29 matrix, $z = -3.31$, $P = 0.001$; 78 matrix, $z = -2.49$, $P = 0.01$)(for SAN-DMN_a, 13 matrix, $z = -1.98$, $P = 0.047$; 29 matrix, $z = -3.05$, $P = 0.002$; 78 matrix, $z = -2.81$, $P = 0.005$)(for the SMN-DMN-VIN, 13 matrix, $z = 2.44$, $P = 0.02$; 29 matrix, $z = 2.31$, $P = 0.02$; 78 matrix, $z = 1.81$, $P = 0.07$). See also Supplementary Note 2. *P* values (for two-tailed testing) were calculated by using generalized linear models. Permutation tests were not performed in these repeated analyses. Significant between-group differences (low-grade inflammatory vs. non-inflammatory groups) in averaged connectivity strength of CEN-DMN_a and SAN-DMN_a were observed across the 3 dimensionalities. Averaged connectivity strength of SMN-DMN-VIN for the low-grade inflammatory group significantly differed from that of the non-inflammatory group in both the 13 X 13 and 29 X 29 matrices. A between-group difference in averaged connectivity strength of SMN-DMN-VIN from the 78 X 78 matrix was also observed, while it is noteworthy that it did not reach statistical significance ($z = 1.81$, $P = 0.07$). Boxes and whiskers indicate the interquartile ranges and the range of data (the 10th and 90th percentiles), respectively. Mean and median values are presented as the red dotted and black lines in the boxes, respectively Source data are provided as a Source Data file.

Abbreviations: CEN, central executive network; SAN, salience network; DMN, default mode network; DMN_a, default mode network, anterior; DMN_p, default mode network, posterior; SMN, sensorimotor network; VIN, visual network.

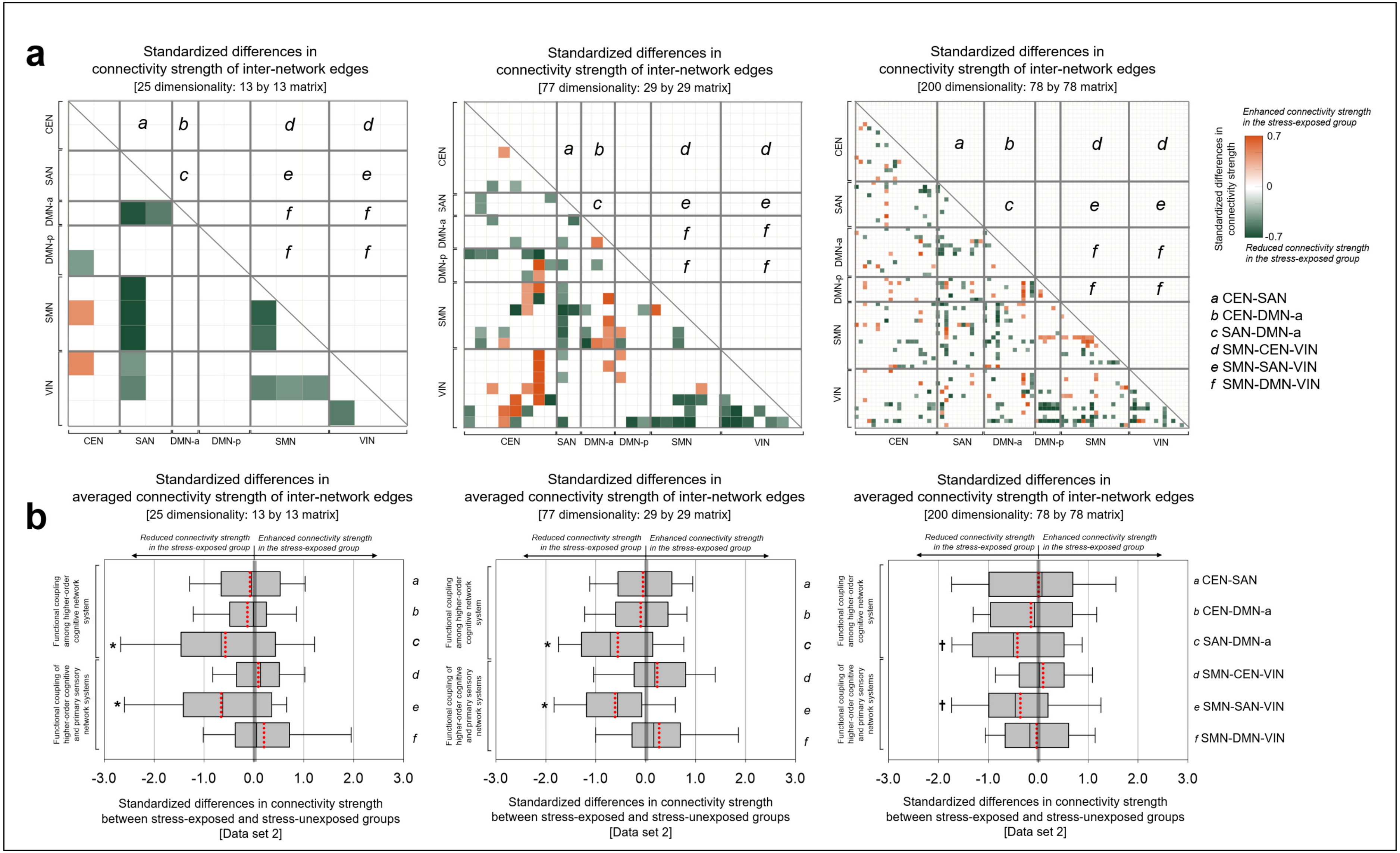

Supplementary Figure 8 Values of matrices with dimensionalities of 25 (left), 77 (automatic estimation, center), and 200 (right) indicating the standardized differences in connectivity strength at an inter-network edge-level between the stress-exposed (n = 52) and stress-unexposed (n = 46) groups of data set 2 at a statistical significance of $P < 0.05$ (**a**) and box and whisker plots representing the distribution of standardized differences in averaged connectivity strength among inter-network edges that connect the network nodes (*a* CEN-SAN; *b* CEN-DMN$_a$; *c* SAN-DMN$_a$; *d* SMN-CEN-VIN; *e* SMN-SAN-VIN; *f* SMN-DMN-VIN) between the stress-exposed (n = 52) and stress-unexposed (n = 46) groups of data

set 2 (**b**).

Standardized between-group differences in connectivity strength were calculated by using the means and standard deviations for inter-network connectivity strength of the stress-unexposed group. Positive and negative values indicate enhanced and reduced connectivity strength, respectively, in the stress-exposed group as compared to the stress-unexposed group. Orange and green colors in the panel a indicate enhanced and reduced connectivity strength in the stress-exposed group relative to the stress-unexposed group, respectively. The composition of inter-network edges, which constitute each block of the matrices (*a* CEN-SAN; *b* CEN-DMN_a; *c* SAN-DMN_a; *d* SMN-CEN-VIN; *e* SMN-SAN-VIN; *f* SMN-DMN-VIN) was similar to, but not same as the composition of inter-network edge clusters derived from the factor analyses in the main findings. Asterisks (*) in panel **b** indicate statistically significant between-group differences in averaged connectivity strength among inter-network edges at $P < 0.05$ (for the SAN-DMN_a,13 matrix, $z = -2.34$, $P = 0.02$; 29 matrix, $z = -2.89$, $P = 0.004$)(for the SMN-SAN-VIN, 13 matrix, $z = -2.92$, $P = 0.003$; 29 matrix, $z = -3.30$, $P = 0.001$). For presentation purposes, a marginal significance at $P < 0.10$ is also marked as a cross (†)(for the SAN-DMN_a, 78 matrix, $z = -1.96$, $P = 0.05$; for the SMN-SAN-VIN, 78 matrix, $z = -1.69$, $P = 0.09$). See also Supplementary Note 2. *P* values (for two-tailed testing) were calculated by using generalized linear models. Permutation tests were not performed in these repeated analyses. Significant between-group differences in averaged connectivity strength of SAN-DMN_a as well as SMN-SAN-VIN were observed across all 3 matrices, while it is noteworthy that it did not reach statistical significance in the case of the 78 X 78 matrix (SAN-DMN_a, $z = -1.96$, $P = 0.05$; SMN-SAN-VIN, $z = -1.69$, $P = 0.09$). Boxes and whiskers indicate the interquartile ranges and the range of data (the 10th and 90th percentiles), respectively. Mean and median values are presented as the red dotted and black lines in the boxes, respectively. Source data are provided as a Source Data file.

Abbreviations: CEN, central executive network; SAN, salience network; DMN, default mode network; DMN_a, default mode network, anterior; DMN_p, default mode network, posterior; SMN, sensorimotor network; VIN, visual network.

Supplementary References

1. Beckmann, C. F. Modelling with independent components. *NeuroImage* **62**, 891-901 (2012).
2. Smith, S. M. et al. Correspondence of the brain's functional architecture during activation and rest. *Proc. Natl. Acad. Sci. U. S. A.* **106**, 13040-13045 (2009).
