# Supplementary material for: A double-hit of stress and low-grade inflammation on functional brain network mediates posttraumatic stress symptoms
Source: Nat Commun. 2020 Apr 20;11:1898. doi: 10.1038/s41467-020-15655-5 (PMC7171097; doi:10.1038/s41467-020-15655-5)
Supplement: Supplementary file 2 — Reporting Summary [file 41467_2020_15655_MOESM2_ESM.pdf]

nature research

Corresponding author(s): In Kyoon Lyoo

Last updated by author(s): Feb 25, 2020

Reporting Summary

Nature Research wishes to improve the reproducibility of the work that we publish. This form provides structure for consistency and transparency in reporting. For further information on Nature Research policies, see Authors & Referees and the Editorial Policy Checklist.

Statistics

For all statistical analyses, confirm that the following items are present in the figure legend, table legend, main text, or Methods section.

n/a	Confirmed	
☐	☒	The exact sample size (*n*) for each experimental group/condition, given as a discrete number and unit of measurement
☐	☒	A statement on whether measurements were taken from distinct samples or whether the same sample was measured repeatedly
☐	☒	The statistical test(s) used AND whether they are one- or two-sided<br>*Only common tests should be described solely by name; describe more complex techniques in the Methods section.*
☐	☒	A description of all covariates tested
☐	☒	A description of any assumptions or corrections, such as tests of normality and adjustment for multiple comparisons
☐	☒	A full description of the statistical parameters including central tendency (e.g. means) or other basic estimates (e.g. regression coefficient) AND variation (e.g. standard deviation) or associated estimates of uncertainty (e.g. confidence intervals)
☐	☒	For null hypothesis testing, the test statistic (e.g. *F*, *t*, *r*) with confidence intervals, effect sizes, degrees of freedom and *P* value noted<br>*Give P values as exact values whenever suitable.*
☒	☐	For Bayesian analysis, information on the choice of priors and Markov chain Monte Carlo settings
☐	☒	For hierarchical and complex designs, identification of the appropriate level for tests and full reporting of outcomes
☒	☐	Estimates of effect sizes (e.g. Cohen's *d*, Pearson's *r*), indicating how they were calculated

Our web collection on statistics for biologists contains articles on many of the points above.

Software and code

Policy information about availability of computer code

Data collection	Brain MRI data were collected using the standard 3.0 T Philips MRI scanner.<br>Peripheral blood inflammatory markers were measured in serum samples using a multiplexed, fluorescent bead-based immunoassay (MILLIPLEX® MAP Human High Sensitivity T Cell Magnetic Bead Panel, Merck Millipore, MA, USA) on a Luminex xMAP® platform (Luminex Corporation, TX, USA). The bead sets were analyzed using a flow-based Luminex™ 200 system (Luminex Corporation, TX, USA). The mean fluorescence intensity of the raw data was captured using the Luminex xPONENT software (Luminex Corporation, TX, USA). The concentrations of the cytokines in each sample were calculated using the MILLIPLEX- Analyst (Viagene Tech, MA, USA) with a five-parameter logistic regression to compute sample concentrations from the standard curves.
Data analysis	Resting-state fMRI data were analyzed using the FMRIB Software Library tools (FSL version 6.0.3, http://www.fmrib.ox.ac.uk/fsl) including MELODIC version 3.15, FIX version 1.06, and FSLNets version 0.6.3.

For manuscripts utilizing custom algorithms or software that are central to the research but not yet described in published literature, software must be made available to editors/reviewers. We strongly encourage code deposition in a community repository (e.g. GitHub). See the Nature Research guidelines for submitting code & software for further information.

Data

Policy information about availability of data

All manuscripts must include a data availability statement. This statement should provide the following information, where applicable:

- Accession codes, unique identifiers, or web links for publicly available datasets
- A list of figures that have associated raw data
- A description of any restrictions on data availability

All relevant data underlying the major findings are available within the manuscript and supplementary information files. Source data underlying Table 1, Fig. 3, 4, and 6, Supplementary Tables 1, 3 through 7, and Supplementary Figures 7 and 8 are provided as a Source Data file. The individual-level data that support the findings of this study are available upon reasonable request from the corresponding author. Due to ethical and legal restrictions, individual-level data of this study cannot be made publicly available. Request to access of individual-level data will be considered in relation to the relevant consents, rules and regulations, and can

be made via the Ewha Brain Institute Review Committee at e600134@ewha.ac.kr after print publication of this paper. Assurance of proper credentials for handling sensitive data will be required from the applicant(s) prior to data sharing. Approval by the Ewha Brain Institute Review Committee will be followed by transfer of data upon signed agreement between Ewha Brain Institute and applicant(s). Applicant(s) may be requested to provide reimbursement of data management or preparation costs, as the Ewha Brain Institute does not receive funding for processes involving data sharing, such as analyses required for the de-identification of data and preparation of protected access.

Field-specific reporting

Please select the one below that is the best fit for your research. If you are not sure, read the appropriate sections before making your selection.

☒ Life sciences ☐ Behavioural & social sciences ☐ Ecological, evolutionary & environmental sciences

For a reference copy of the document with all sections, see nature.com/documents/nr-reporting-summary-flat.pdf

Life sciences study design

All studies must disclose on these points even when the disclosure is negative.

Sample size	Sample size of the MRI and inflammatory markers was based on data availability. No statistical methods were used to predetermine the sample size.
Data exclusions	Data exclusion criteria for all subjects were pre-established. The exclusion criteria of the subjects for data set 1 were 1) significant medical, neurological or psychiatric disorders as assessed using the medical, neurological, and psychiatric history taking including the assessment through the Structured Clinical Interview for DSM-4 and routine laboratory tests, 2) significant structural abnormalities as found in the brain magnetic resonance imaging scans confirmed by an experienced neuroradiologist, 3) having experienced any type of serious traumatic event, 4) a history of severe traumatic brain injury, or 5) any contraindications to MRI. For data set 2, those who met the diagnostic criteria for PTSD were excluded from the study, and only those with subclinical levels of PTS symptoms were included as part of the stress-exposed group for further analysis. Other than the exclusion criterion regarding trauma exposure, the inclusion and exclusion criteria applied to individuals in data set 2 were identical to those of data set 1. For the neuroimaging data, failure to meet adequate data quality was also an exclusion criterion.
Replication	The findings of this study were replicated in a series of sensitivity analyses presented in Supplementary Information. We conducted sensitivity analyses by including the additional covariates of head motion or body mass index in the generalized linear model in both data sets 1 and 2. Results of these four analyses were similar to those from the original model (Supplementary Note 1). In addition, group ICA was repeated to demonstrate the consistency of inter-network functional connectivity patterns across a range of dimensionality, which were the automatic estimation of optimal dimensionality and a predetermined dimensionality of 200. Results from these 2 additional analyses demonstrated that inter-network functional connectivity patterns are consistent among various dimensionality settings (Supplementary Note 2).
Randomization	Not applicable. Participants were divided into groups according to pre-defined criteria.
Blinding	Blinding was not relevant to this study since the participants in each group had different characteristics regarding inflammatory markers (data set 1) or a history of life-threatening trauma exposure (data set 2).

Reporting for specific materials, systems and methods

We require information from authors about some types of materials, experimental systems and methods used in many studies. Here, indicate whether each material, system or method listed is relevant to your study. If you are not sure if a list item applies to your research, read the appropriate section before selecting a response.

Materials & experimental systems

n/a	Involved in the study	
☒	☐	Antibodies
☒	☐	Eukaryotic cell lines
☒	☐	Palaeontology
☒	☐	Animals and other organisms
☐	☒	Human research participants
☒	☐	Clinical data

Methods

n/a	Involved in the study	
☒	☐	ChIP-seq
☒	☐	Flow cytometry
☐	☒	MRI-based neuroimaging

Human research participants

Policy information about studies involving human research participants

Population characteristics	We used two independent data sets collected in South Korea. Study participants in data set 1 were healthy adult volunteers (mean [standard deviation, SD], 32.7 [11.6] years; 410 men and 290 women). Data set 2 (n = 98) consists of 52 firefighters who have been repeatedly exposed to direct and/or indirect forms of life-threatening traumatic events (the stress-exposed group,

	mean [SD], 33.6 [4.0] years; 45 men and 7 women) and 46 healthy individuals who were not exposed to any significant traumatic event (the stress-unexposed group, mean [SD], 33.0 [3.8] years; 39 men and 7 women).
Recruitment	Healthy participants in data set 1 were recruited through community advertisement. Firefighters in data set 2 were recruited at fire stations within the Seoul metropolitan area of South Korea, while the demographically matched healthy individuals were recruited through community advertisement within the Seoul metropolitan area. Potential sampling biases that might be present in this study were addressed as a limitation in the Discussion section of the manuscript.
Ethics oversight	The Institutional Review Board of Ewha W. University approved the study protocol and all participants provided written informed consent. All of the study processes were in alignment with the Declaration of Helsinki as well as national and institutional regulations and guidelines.

Note that full information on the approval of the study protocol must also be provided in the manuscript.

Magnetic resonance imaging

Experimental design

Design type	Resting-state functional magnetic resonance imaging
Design specifications	The duration of each resting-state fMRI run was approximately 8 minutes.
Behavioral performance measures	No behavioral measures were acquired during the fMRI scan recordings.

Acquisition

Imaging type(s)	Functional magnetic resonance imaging with high-resolution structural imaging (T1-weighted imaging)
Field strength	3 Tesla
Sequence & imaging parameters	Structural brain imaging data were collected using a T1-weighted magnetization-prepared rapid gradient echo imaging sequence with the following parameters: echo time, 3.4 ms; repetition time, 7.4 ms; flip angle, 8º; field of view, 220 X 220 mm^2; slice thickness, 1 mm; 180 contiguous sagittal slices.<br>Resting-state functional MRI (fMRI) scans were performed using an echo planar imaging sequence with the following acquisition parameters: echo time, 21 ms; repetition time, 2,000 ms; flip angle, 76º; field of view, 220 X 220 mm^2; slice thickness, 3.5 mm; 200 volumes; 38 slices.
Area of acquisition	Whole brain

Diffusion MRI ☐ Used ☒ Not used

Preprocessing

Preprocessing software	FMRIB Software Library tools (FSL, http://www.fmrib.ox.ac.uk/fsl)
Normalization	Resting-state fMRI data image of each individual was first co-registered to the corresponding high-resolution T1-weighted image. These co-registered images were linearly registered to the standard Montreal Neurological Institute (MNI) 152 template with 12 degrees of freedom, subsequently resampled to 4-mm isotropic voxel space.
Normalization template	MNI 152
Noise and artifact removal	A data-driven denoising strategy was employed to remove head motion and structural artifacts at an individual image level using single-subject ICA implemented by Multivariate Exploratory Linear Optimized Decomposition into Independent Components (MELODIC) followed by FMRIB's ICA-based Xnoiseifier (FIX).
Volume censoring	As one of the pre-established exclusion criteria, the images with mean framewise displacement of greater than or equal to 0.3 mm were excluded from the study.

Statistical modeling & inference

Model type and settings	In order to generate a set of group-averaged network nodes, a model-free and data-driven approach of group ICA with a dual regression algorithm was applied to decompose the preprocessed four-dimensional fMRI images of all 798 individuals from data sets 1 and 2 into a set of three-dimensional spatial maps and one-dimensional time series. A total of 13 resting-state networks (RSNs) were selected and thresholded at $z = 3$ ($P = 0.001$) and used as "network nodes" for constructing internetwork connection matrix. The strength of inter-network functional edges were measured to examine the neurobiological correlates of low-grade inflammation and stress exposure, and the 13 X 13 correlation matrix was then generated for each subject as to represent correlations between all pairs of network nodes. These connectivity values were transformed into "inter-network connectivity strength" by multiplying inter-network connectivity values with the sign of their mean connectivity value.
Effect(s) tested	For data set 1, generalized linear models were performed to examine differences in standardized mean connectivity strength of each inter-network connection cluster between the low-grade inflammatory and non-inflammatory groups after adjusting for age and sex. For data set 2, differences in standardized mean connectivity strength of each

internetwork cluster were examined between the low-grade inflammatory and non-inflammatory groups as well as between the stress-exposed and stress-unexposed groups, respectively, using generalized linear models.

Specify type of analysis: ☒ Whole brain ☐ ROI-based ☐ Both

Statistic type for inference
(See Eklund et al. 2016)

Network nodes in this study were based on the voxel-wise group ICA using FSL Randomise.

Correction

Permutation-adjusted P values for each model were calculated. A total of 10,000 permutations were performed to obtain an empirical null distribution of effects under the null-hypothesis.

Models & analysis

n/a	Involved in the study	
☐	☒	Functional and/or effective connectivity
☒	☐	Graph analysis
☒	☐	Multivariate modeling or predictive analysis

Functional and/or effective connectivity

Pearson correlation
